# *KIR* and *HLA-C* genes in male infertility

**DOI:** 10.1007/s10815-020-01814-6

**Published:** 2020-05-20

**Authors:** Karolina Wilczyńska, Paweł Radwan, Rafał Krasiński, Michał Radwan, Jacek R. Wilczyński, Andrzej Malinowski, Ewa Barcz, Izabela Nowak

**Affiliations:** 1 Department of Clinical Immunology, Laboratory of Immunogenetics and Tissue Immunology, Ludwik Hirszfeld Institute of Immunology and Experimental Therapy, Polish Academy of Sciences, Wrocław, Poland; 2 Department of Reproductive Medicine, Gameta Hospital, Rzgów, Poland; 3 Faculty of Health Sciences, The State University of Applied Sciences in Płock, Płock, Poland; 4 Department of Surgical and Oncological Gynecology, Medical University of Łódź, Łódź, Poland; 5 Department of Surgical, Endoscopic and Oncologic Gynecology, Polish Mothers’ Memorial Hospital–Research Institute, Łódź, Poland; 6 First Chair and Clinic of Obstetrics and Gynecology, Medical University of Warsaw, Warszawa, Poland

**Keywords:** KIR, HLA-C polymorphism, In vitro fertilization embryo transfer, Infertility, Semen, Sperm quality and recurrent spontaneous abortion

## Abstract

**Purpose:**

Approximately 50% of men reporting to clinics for assisted reproduction have abnormal sperm parameters; we therefore considered whether they differ from fertile males in terms of the frequency of *KIR* and *HLA-C* genes, suggesting the involvement of NK cells and some T cells in the inflammatory reaction that can occur in the testes, vas deferens, or epididymis.

**Method:**

We tested a total of 1064 men: 445 of them were patients who, together with their female partners, participated in in vitro fertilization (IVF), 298 men whose female partners suffered from recurrent spontaneous abortion. Three hundred twenty-one fertile men constituted the control group. *KIRs* were genotyped using KIR Ready Gene kits and *HLA-C* by PCR-SSP methods.

**Results:**

We found differences in *KIR* gene frequencies between men who became fathers via natural conception and men who participated in in vitro fertilization for *KIR2DL2* (*p/p*_*corr.*_ = 0.0015/0.035, OR = 1.61), *KIR2DL5 gr.2* (*p/p*_*corr.*_ = 0.0023/0.05, OR = 1.64), *KIR2DS2* (*p/p*_*corr*._ = 0.0019/0.044, OR = 1.59), and *KIR2DS3* (*p/p*_*corr*._ = 0.0016/0.037, OR = 1.67). *KIRs* in Cen AA region were significantly overrepresented in fertile males than in IVF males (*p/p*_*corr*._ = 0.0076/0.03, OR = 0.67), whereas Cen AB + Cen BB frequency was higher in IVF males than in fertile males (*p/p*_*corr*._ = 0.0076/0.03, OR = 1.50). We also observed a limited association in KIR-HLA-C combinations.

**Conclusion:**

Fertile men differ in profile of *KIR* genes and *KIR-HLA-C* combinations from men participating in IVF.

**Electronic supplementary material:**

The online version of this article (10.1007/s10815-020-01814-6) contains supplementary material, which is available to authorized users.

## Introduction

Male factors, either alone or in combination with female causes, contribute to infertility in approximately 50% of couples who fail to conceive. This is a growing problem in the world, as well as in Poland. A decrease in the quality of semen has been observed over the years and may be caused by endocrine disrupting chemicals [1–4]. Male infertility can also result from anatomical or genetic abnormalities, systemic or neurological diseases, infections, trauma, iatrogenic injury, gonadotoxins, and development of antisperm antibodies. Male infertility may be due to testicular and post-testicular deficiencies. However, in 30–40% of male infertility cases, no cause is identified, this condition is named: idiopathic male infertility [5]. Moreover, male factors may have an influence on fertilization and embryo development failure, an increase in the risk of idiopathic recurrent miscarriages, autosomal dominant diseases, and neurobehavioral disorders in offspring [6].

Since the role of inflammatory response and infection is postulated in male infertility, and thus also the participation of natural killer (NK) cells and T lymphocytes, perhaps the role of killer immunoglobulin-like receptor (KIR) and its ligands may be presumed. KIR receptors play a central role in the control of NK cell function, but may also be expressed by some lymphocytes [7–10]. KIR can be of inhibitory or activating function. Inhibitory KIRs have cytoplasmic domains with immunoreceptor tyrosine-based inhibitory (ITIM) motifs which inhibit NK function, whereas activating KIRs contain positively charged lysine residues in the transmembrane region and have truncated cytoplasmic tails lacking ITIMs. Ligation of activating KIR leads to recruitment of the protein DAP12 with immunoreceptor tyrosine-based activation motif (ITAM), which initiates intracellular signaling pathways and induces NK cell activation [11, 12]. The KIR consists of two (KIR2D) or three (KIR3D) extracellular immunoglobulin-like domains, a stem region, a transmembrane domain, and a cytoplasmic tail. Typically, inhibitory KIRs possess a long cytoplasmic tail (e.g., KIR2DL) while activating KIRs possess a short cytoplasmic tail (e.g., KIR2DS). KIR2DL4 is the one exception; it has a long cytoplasmic tail but activates NK cells [13].

*KIR* genes, encoded by chromosome 19, show extensive polymorphism. People differ in both the number and kinds of *KIRs*. This results in differences in the frequencies of these genes between populations. *KIRs* can be organized into two haplotypes: A and B. Both haplotypes consist of four framework genes: *KIR3DL3* at the centromeric end, *KIR3DL2* at the telomeric end, and *KIR3DP1* and *KIR2DL4* in the middle of the *KIR* gene cluster. Group A haplotypes are characterized by possessing *KIR2DL1*, *KIR2DL3*, *KIR3DL1*, *KIR2DS4*, and *KIR2DP1* and exert inhibition of cell function despite the presence of *KIR2DS4*. This gene in most Caucasians occurs as a deletion variant that encodes a nonfunctional receptor (KIR2DS4-del). Group B haplotypes show variation in the number and combination of *KIR* genes. They may possess one or more of *KIR2DL2*, *KIR2DL5A/B*, *KIR2DS1*, *KIR2DS2*, *KIR2DS3*, *KIR2DS5*, and *KIR3DS1* genes and may exert activating function upon interaction with an appropriate ligand [14]. Individual who carries 2 copies of haplotype A is considered as the AA genotype, and man who has haplotype B genes is considered as the Bx genotype (AB + BB). NK cell function depends on the balance between activating and inhibitory receptors. Thus, the AA genotype has a set of genes for the largest number of inhibitory receptors, with no functional activating genes. The inhibition intensity decreases as the number and expression of genes for activating KIR receptors increases.

The ligands for the inhibitory and activating KIRs constitute HLA-A, HLA-B, and HLA-C allotypes. For some activating KIRs: 2DS3, 2DS5 the ligand is not known due to the lack of specific antibodies. Antibodies that bind to the activating receptor also bind to its inhibitory counterpart, because of very high homology of extracellular domains [15].

*HLA-C* gene is enormously polymorphic [16]. *HLA-C* alleles occur in two allotypes, C1 and C2, based on the presence of asparagine or lysine at position 80 of the HLA-C α-domain, respectively. HLA-C C1 allotypes bind inhibitory KIR2DL2/3, while HLA-C C2 allotypes bind KIR2DL1 and KIR2DS1. However, the interaction of the former is weaker than KIR2DL1-HLA-C C2. Other KIR-ligand pairs were presented in a review article by Nowak et al. [17].

Approximately 50% of men reporting to clinics for assisted reproduction have abnormal sperm parameters; we therefore considered whether they differ from fertile males in terms of the frequency of *KIR* and *HLA-C* genes, suggesting the involvement of NK cells and some T cells in the inflammatory reaction that can occur in the testes, vas deferens, or epididymis. Secondly, can these tested genes be susceptibility genes for male infertility. Thirdly, which of potential KIR genes/receptors and *KIR* – *HLA-C* combinations could affect semen parameters. There is no literature data that would indicate the participation of KIR receptors, their ligands, and their genes in male infertility. The only published study concerns cryptorchidism in the Polish population and potential participation of *KIR* genes in susceptibility to this disease [18].

## Materials and methods

### Study design

**Fig. 1:**
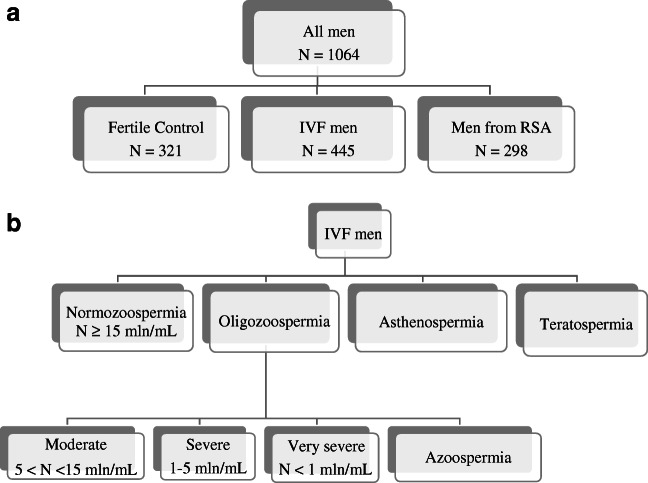
Study population flowchart. **a**) The number and division into groups of men tested in this study. **b**) Division of IVF men by semen parameters

In our research, we tested a total of 1064 men. Four hundred and forty-five males were patients who, together with their female partners, participated in in vitro fertilization (IVF), while a group of 321 fertile men constituted the control group (Fig. 1a). Patients were qualified at the Gameta Assisted Reproduction Clinic in Rzgów, the first center in Europe certified by the European Society for Human Reproduction and Embryology (ESHRE ART Center Certification for good clinical practice). Patients were also recruited from the Department of Surgical, Endoscopic, and Oncologic Gynecology and Department of Gynecology and Gynecologic Oncology, Polish Mothers’ Memorial Hospital–Research Institute in Łódź and Gynemed. The men were of mean age 36.27 years ± 4.97 (age range 24–53). Patients were included in the studies from April 2015 to February 2020. Another group studied in this work was a group of men whose partners suffered from recurrent spontaneous abortion (RSA men). They were of mean age 34.06 ± 3.21 years (age range 27–41). These men were also recruited from the Department of Surgical, Endoscopic, and Oncologic Gynecology and Department of Gynecology and Gynecologic Oncology, Polish Mothers’ Memorial Hospital–Research Institute, Poland. Material from these men was collected in the years 2006–2014. The clinical characteristics of couples who participated in in vitro fertilization are presented in Table 1.

**Table 1:** Clinical characteristics of couples participated in IVF-ET

	All patients *N* = 445	Recurrent implantation failure *N* = 269	Successful pregnancy *N* = 141	Patients unclassified *N* = 35
Female age, mean ± SD; range	33.70 ± 4.12; 22–46	34.51 ± 4.13; 23–46	32.09 ± 3.73; 22–41	34.03 ± 3.64; 27–45
Male age, mean ± SD; range	36.27 ± 4.97; 24–53	36.27 ± 4.86; 25–53	34.37 ± 4.89; 24–53	37.13 ± 4.90; 28–51
Number of IVF-ET, mean ± SD; range	3.34 ± 1.95; 1–15	4.42 ± 1.73; 3–15	1.62 ± 0.69; 1–3	1.73 ± 0.45; 1–2
Number of all transferred embryos, mean ± SD; range	3.79 ± 2.42; 1–19	5.09 ± 2.23; 3–19	1.66 ± 0.72; 1–3	1.85 ± 0.54; 1–3
Type of IVF procedure (%):				
ICSI	350 (78.65)	191 (71.04)	136 (96.45)	16 (45.71)
IMSI	22 (4.94)	10 (3.72)	11 (7.80)	1 (2.86)
IVF	85 (19.10)	70 (26.02)	7 (4.96)	7 (20.00)
FET	258 (57.98)	156 (57.99)	85 (60.28)	12 (34.29)
Indications for IVF (%):				
Only male factor	137 (30.79)	75 (27.88)	55 (39.01)	6 (23.08)
Only female factor	113 (25.39)	65 (24.16)	37 (26.24)	8 (30.77)
Both factors	67 (15.06)	44 (16.36)	16 (11.35)	5 (19.23)
Unexplained infertility	128 (28.76)	85 (31.60)	33 (23.40)	7 (26.92)

For unclassified patients, we had clinical data only for 26 couples

73 patients from the RIF group finally became pregnant in subsequent embryo transfers and gave birth to a child

The control group was qualified mainly from the 1st Department of Obstetrics and Gynecology, Medical University of Warsaw in the years 2006–2014. These men and their female partners had at least one healthy child from natural conception. The men were of mean age 34.07 years ± 6.19 (age range 25–70). All tested males were of Polish origin. Men from the control group differed in age from IVF men (*p* < 0.0001), but not with men from RSA group (*p* = 0.2).

### Procedures pre-IVF for qualification of patients

During the qualification of men for IVF, they were ordered to check for infections: hepatitis B surface antigen (HBsAg), anti-HCV, anti-HIV½ and syphilis antibody (VDRL: Venereal Disease Research Laboratory), CMV IgM, CMV IgG. Men with bad sperm parameters had consultations with the urologist and performed testicular ultrasound. If the symptoms of genital infection were diagnosed, patient was treated according to antibiogram and The European Association of Urology Guidelines.

### Semen analysis

The semen samples were obtained by masturbation after 2–7 days of sexual abstinence. The patients’ ejaculate samples were analyzed and categorized according to the nomenclature of the WHO (World Health Organization) from 2010 [19]. Normozoospermia means total number of sperm cells, their concentration, and progressive motility and morphology above or equal reference values (*N* ≥ 15 mln/mL of sperm cells); OS, oligozoospermia; moderate OS (5 < *N* < 15 mln/mL), severe OS (1–5 mln/mL), very severe OS (*N* < 1 mln/mL); AS, azoospermia (lack of sperm cells in ejaculate); asthenospermia, number of sperm cells with progressive motility below reference values; and teratospermia, number of morphologically normal sperm cells below reference values (Fig. 1b).

### DNA preparation and genotyping

Genomic DNA was isolated from venous blood using the Invisorb Spin Blood Midi Kit (Invitek, Berlin, Germany) or Qiagen (Germany) according to the manufacturer’s instructions.

*KIRs* were genotyped using KIR Ready Gene kits (Inno-train Diagnostics, Germany) following the manufacturer’s instructions (for details see Supplementary Fig. 1 and Supplementary Table 1), or multiplex PCR described elsewhere [20, 21]. Our *KIR* typing is validated three times per year by the International KIR Exchange program organized by the Immunogenetics Center of the University of California, Los Angeles. *KIR* AA genotype means the presence of *KIR2DL1*, *KIR2DL3*, *KIR2DS4*, and *KIR3DL1*, and the absence of *KIR2DL5*, *KIR2DS1*, *KIR2DS2*, *KIR2DS3*, *KIR2DS5*, and *KIR3DS1*, which may be found in KIR Bx genotype. *KIRs* were also divided according to presence in the centromeric or telomeric part of the *KIR* gene cluster: CenA-KIR2DL3, CenB-KIR2DL2 and KIR2DS2, TelA-KIR3DL1 and KIR2DS4, TelB-KIR2DS1 and KIR3DS1 [22].

*HLA-C* gene fragments determining the C1 and C2 allotypes were detected in a PCR-SSP method described in detail elsewhere [21, 23].

### Statistical analysis

For the analysis of *KIR* and *HLA-C* genotype frequencies, we used the two-tailed Fisher exact test (GraphPad Prism 5 software). Hardy-Weinberg equilibrium was estimated using the chi-square test with one degree of freedom. All genotype frequencies were in Hardy-Weinberg equilibrium both in control and in patient groups. A *p* value < 0.05 was considered significant. The odds ratio (OR) and its 95% confidence interval (95% CI) were computed as the measure of effect size. For multiple comparison tests, Bonferroni correction was done.

## Results

### Comparison of profile of *KIR* genes in fertile men vs. men participating in IVF and fertile men vs. recurrent spontaneous abortion

We found differences in *KIR* gene frequencies between men who became fathers’ children via natural conception and men who participated in in vitro fertilization (Table 2). Differences were significant even after correction for multiple comparisons for *KIR2DL2* (*p/p*_*corr*._ = 0.0015/0.035, OR = 1.61), *KIR2DL5 gr.2* (*p/p*_*corr*._ = 0.0023/0.05, OR = 1.64), *KIR2DS2* (*p/p*_*corr*._ = 0.0019/0.044, OR = 1.59), and *KIR2DS3* (*p/p*_*corr*._ = 0.0016/0.037, OR = 1.67). When we divided *KIRs* according to their presence in the centromeric or telomeric part of the *KIR* gene cluster, we observed that *KIRs* in cen AA region were significantly overrepresented in fertile males than in IVF males (*p/p*_*corr*._ = 0.0076/0.03, OR = 0.67), while Cen AB + Cen BB frequency was higher in IVF males than in fertile males. In addition, Cen AA/Tel AA genotype was more prevalent in fertile men in comparison with IVF men (*p/p*_*corr*._ = 0.041/ns, OR = 0.71), whereas Cen AB/Tel BB in IVF men (*p/p*_*corr*._ = 0.054/ns, OR = 2.66; Table 3). We also analyzed the profile of *KIR* genes in men from the RSA group. Results were shown in Supplementary Table 2. We also found a higher frequency of *KIR2DL2*, *KIR2DL5*, *KIR2DS2*, and *KIR2DS3* genes in men from RSA group than in control males; however, this comparison was not statistically significant (*p* = 0.08, OR = 1.33 for *KIR2DL2*; *p* = 0.09, OR = 1.32 for *KIR2DL5*; *p* = 0.06, OR = 1.35 for *KIR2DS2*; *p/p*_*corr*._ = 0.03/ns, OR = 1.47 for *KIR2DS3*; Supplementary Table 2).

**Table 2:** *KIR* gene frequencies in fertile men and men who participated in IVF

KIR	Fertile control *N* = 321	IVF men N = 445	IVF men vs. fertile control		
			*P*/*P*_corr._	OR	95%CI
2DL1	311 (97.19)	422 (94.83)	0.21	0.59	0.28–1.26
2DL2	158 (49.22)	271 (60.90)	*0.0015/0.035*	*1.61*	*1.2–2.15*
2DL3	290 (90.34)	385 (86.52)	0.11	0.69	0.43–1.09
2DL4 norm	212 (66.04)	313 (70.34)	0.21	1.22	0.90–1.66
2DL4 del	250 (77.88)	347 (77.98)	1.00	1.01	0.71–1.42
2DL5 all	145 (45.17)	232 (52.13)	0.067	1.32	0.99–1.76
2DL5 gr.1	77 (23.99)	119 (26.74)	0.40	1.16	0.83–1.61
2DL5 gr.2	85 (26.48)	165 (37.08)	*0.0023/0.05*	*1.64*	*1.20–2.24*
2DL5 exp	103 (32.09)	164 (36.85)	0.19	1.24	0.91–1.67
2DL5 null	98 (30.53)	176 (39.55)	*0.012/ns*	*1.49*	*1.10–2.02*
2DS1	117 (36.45)	170 (38.20)	0.65	1.08	0.80–1.45
2DS2	159 (49.53)	271 (60.90)	*0.0019/0.044*	*1.59*	*1.19–2.12*
2DS3	82 (25.55)	162 (36.40)	*0.0016/0.037*	*1.67*	*1.22–2.29*
2DS4 norm	105 (32.71)	159 (35.73)	0.40	1.14	0.84–1.55
2DS4 del	271 (84.42)	366 (82.25)	0.44	0.85	0.58–1.26
2DS5	77 (23.99)	118 (26.52)	0.45	1.14	0.82–1.59
3DL1	303 (94.39)	416 (93.48)	0.65	0.85	0.46–1.56
3DL2	321 (100.0)	443 (99.56)	0.51	0.28	0.01–5.77
3DL3	321 (100.0)	445 (100.0)	–	–	–
3DS1	107 (33.33)	165 (37.08)	0.32	1.18	0.87–1.59
2DP1	314 (97.82)	424 (95.28)	0.079	0.45	0.19–1.07
3DP1	314 (97.82)	422 (94.83)	*0.038/ns*	*0.41*	*0.17–0.97*
3DP1 var	102 (31.78)	158 (35.51)	0.31	1.18	0.87–1.60

Values in italics indicate significant differences. Values in parentheses are in percentages

*IVF* in vitro fertilization; *P* probability; *P*_corr._ *P* × 23 tested variants—Bonferroni correction for multiple comparisons; *OR* odds ratio; *95*% *CI* confidence interval from two-sided Fisher’s exact test; *ns* not significant

**Table 3:** Centromeric and telomeric *KIR* genotypes in fertile men and men who participated in IVF

KIR genotype	Fertile control *N* = 321	IVF men *N* = 445	IVF men vs. fertile control		
			*P*/*P*_corr._	OR	95%CI
AA	102 (31.78)	111 (24.94)	*0.041/ns*	*0.71*	*0.52–0.98*
Bx	219 (68.22)	334 (75.06)	*0.041/ns*	*1.40*	*1.02–1.93*
Cen AA	155 (48.29)	171 (38.43)	*0.0076/0.03*	*0.67*	*0.50–0.89*
Cen AB	135 (42.05)	214 (48.09)	0.11	1.28	0.96–1.71
Cen BB	31 (9.66)	60 (13.48)	0.11	1.46	0.92–2.31
Cen AB + Cen BB	166 (51.75)	274 (61.57)	*0.0076/0.03*	*1.50*	*1.12–2.00*
Tel AA	197 (61.37)	263 (59.10)	0.55	0.91	0.68–1.22
Tel AB	111 (34.58)	157 (35.28)	0.88	1.03	0.76–1.39
Tel BB	13 (4.05)	25 (5.62)	0.4	1.41	0.71–2.80
Tel AB + Tel BB	124 (38.63)	182 (40.90)	0.55	1.10	0.82–1.48
Cen AA/Tel AA	102 (31.78)	111 (24.94)	*0.041/ns*	*0.71*	*0.52–0.98*
Cen AA/Tel AB	49 (15.26)	57 (12.81)	0.34	0.82	0.54–1.23
Cen AA/Tel BB	4 (1.25)	3 (0.67)	0.46	0.54	0.12–2.42
Cen AB/Tel AA	80 (24.92)	117 (26.29)	0.67	1.08	0.77–1.49
Cen AB/Tel AB	50 (15.58)	79 (17.75)	0.44	1.17	0.79–1.72
Cen AB/Tel BB	5 (1.55)	18 (4.05)	*0.054/ns*	*2.66*	*0.98–7.25*
Cen BB/Tel AA	15 (4.67)	35 (7.87)	0.10	1.74	0.93–3.25
Cen BB/Tel AB	12 (3.74)	21 (4.72)	0.59	1.28	0.62–2.63
Cen BB/Tel BB	4 (1.25)	4 (0.90)	0.73	0.72	0.18–3.00

Values in italics indicate significant differences. Values in parentheses are in percentages

*IVF* in vitro fertilization; *P* probability; *P*_corr_. *P* × 4—Bonferroni correction for multiple comparisons; *OR* odds ratio; *95*% *CI* confidence interval from two-sided Fisher’s exact test; *ns* not significant

### Potential interactions between associated *KIRs* and *HLA-C* allotypes

Although we did not detect differences in *HLA-C* genotype frequencies, such differences were noticeable in possible KIR-ligand interactions, which were presented in Supplementary Tables 3 and 4. Both IVF carriers of the particular KIR-C1 or KIR-C2 combination differed from fertile men. This effect was manifested in the combination of HLA-C heterozygotes with *KIRs 2DL2*, *2DL5 gr.2*, *2DS2*, and *2DS3* and gave significant differences with odds ratios ranging from 1.54–1.82 and *p* values significant after correction for multiple comparisons (Supplementary Table 3). After comparing the frequency of combinations of *KIRs* in centromeric and telomeric regions with *HLA-C* allotypes between analyzed male groups, we must conclude that after correcting for multiple comparisons, only two of them remained significant. This happens when there are too few numbers in the analyzed groups. However, it should be emphasized that the biggest difference between fertile men and those who participated in IVF was recorded for Cen AA/C2+ (*p/p*_*corr*._ = 0.0058/0.029, OR = 0.64; Supplementary Table 4). This combination was more frequent in fertile men. Moreover, male carriers of the Cen AB/Tel BB/C2+ combination from IVF group were more frequent than control males (*p/p*_*corr*._ = 0.01/0.05, OR = 5.56; Supplementary Table 4). Since only 2 men from the control group and 15 men from IVF group were carriers of such a combination, it would be reasonable to increase the group of men tested to confirm the odds ratio of this analysis. Nevertheless, we can conclude that AA genotype in combination with HLA-C2+ protects from infertility whereas genotypes with activating *KIRs* (particularly Cen AB/Tel BB/C2+) predispose. For the RSA group and control males, we detected differences in frequencies of KIR2DL2/C1+ (*p* = 0.06, OR = 1.36), KIR2DL5/C1+ (*p* = 0.09, OR = 1.34), KIR2DS2/C1+ (*p* = 0.05, OR = 1.38), KIR2DS3/C1+ (*p* = 0.03, OR = 1.53), and KIR2DS4 norm/C1C2 combinations (*p* = 0.02, OR = 1.60; Supplementary Table 2). Unfortunately, significant results disappeared after correction for multiple comparisons. However, the RSA group is almost twice smaller than the IVF group.

We can conclude that KIR-HLA-C activating interactions may affect male infertility and, indirectly, couple infertility.

### *KIR* and *HLA-C* genes in IVF men stratified according to sperm parameters

To check whether *KIR* and *HLA-C* genes affect sperm parameters, we compared the *KIR* profile and *HLA-C* genotype frequencies in IVF men with different sperm abnormalities. We found that IVF men with moderate oligozoospermia possessed a higher frequency of *KIR2DL3*, *KIR2DL5* gr.1, and *KIR2DS5* than IVF men with normozoospermia (*p/p*_*corr*._ = 0.043/ns, OR = 2.47; *p/p*_*corr*._ = 0.023/ns, OR = 1.87; *p/p*_*corr*._ = 0.044/ns, OR = 1.78; respectively; Supplementary Table 5). In addition, normospermic IVF men were with a higher incidence of *KIRs* in the Cen BB region in comparison with IVF men with moderate oligozoospermia (*p/p*_*corr*._ = 0.043/ns, OR = 0.04; Supplementary Table 6). However, these associations were weak. *KIR* genes do not affect susceptibility to severe and very severe oligozoospermia or the absence of sperm in ejaculate. We also observed no association of particular *KIR* genes with asthenozoospermia or teratozoospermia (Supplementary Table 7). However, asthenospermic and teratospermic men were more frequent carriers of Cen AB than normospermic (*p/p*_*corr*._ = 0.046/ns, OR = 1.61; *p/p*_*corr*._ = 0.015/ns, OR = 2.82, respectively; Supplementary Table 8). Since significant results have lost their significance after Bonferroni correction, we cannot conclude that KIRs may have an impact on reducing sperm count.

## Discussion

In this work, we managed to show significant differences in the profile of *KIR* genes between men who have children from natural conception and men who participated with their female partners in an in vitro fertilization. A relatively large group of IVF and control men tested, almost 770, were also enough for the analyses in which we considered the participation of the combination of *KIR* and *HLA-C* genes. Similar results were also observed for men from the RSA group; however, the number of tested individuals (*N* = 298) in this group was not sufficient to be significant after corrections for multiple comparisons. To make the results easier to interpret and discuss in the light of the available literature, we have compiled a summary of relevant results in Table 4. To date, no studies have been published on the role of KIR and the interaction of KIR-HLA-C genes with male infertility. The only published study concerns cryptorchidism in the Polish population and potential participation of *KIR* genes in susceptibility to this disease. Results from that study suggest that KIR2DL2+/KIR2DS2+ genotype may be protective against cryptorchidism [18]. However, in that study, relatively small groups of boys with cryptorchidism (*N* = 109) and young blood donors (*N* = 136) were tested.

**Table 4:** Summarized effect of *KIR* and *HLA-C* polymorphisms on susceptibility to male infertility

KIR and/or HLA-C combination	Associated combination	*P/ P*_*corr.*_	OR	95% CI	Effect	Table
2DL2	IVF vs. fertile control	*0.0015/0.035*	1.61	1.2–2.15	**↑**	1
2DL5 gr.2	IVF vs. fertile control	*0.0023/0.05*	1.64	1.20–2.24	**↑**	1
2DL5 null	IVF vs. fertile control	0.012/ns	1.49	1.10–2.02	↑	1
2DS2	IVF vs. fertile control	*0.0019/0.044*	1.59	1.19–2.12	**↑**	1
2DS3	IVF vs. fertile control	*0.0016/0.037*	1.67	1.22–2.29	**↑**	1
3DP1	IVF vs. fertile control	0.038/ns	0.41	0.17–0.97	↓	1
2DL2	RSA vs. fertile control	0.077	1.33	0.97–1.83	↑	S2
2DL5	RSA vs. fertile control	0.09	1.32	0.96–1.81	↑	S2
2DS2	RSA vs. fertile control	0.06	1.35	0.99–1.86	↑	S2
2DS3	RSA vs. fertile control	0.03/ns	1.47	1.04–2.08	↑	S2
2DL2/C1+	RSA vs. fertile control	0.06	1.36	0.99–1.87	↑	S2
2DL5/C1+	RSA vs. fertile control	0.09	1.34	0.97–1.84	↑	S2
2DS2/C1+	RSA vs. fertile control	0.05/ns	1.38	1.01–1.89	↑	S2
2DS3/C1+	RSA vs. fertile control	0.03/ns	1.53	1.06–2.20	↑	S2
2DS4norm/C1C2	RSA vs. fertile control	0.02/ns	1.60	1.07–2.40	↑	S2
2DL2/C1+	IVF vs. fertile control	*0.0026/0.013*	*1.57*	*1.18–2.10*	**↑**	S3
2DL2/C2+	IVF vs. fertile control	*0.0029/0.015*	*1.60*	*1.18–2.17*	**↑**	S3
2DL2/C1C2	IVF vs. fertile control	*0.0026/0.013*	*1.65*	*1.19–2.30*	**↑**	S3
2DL5 gr.2/C1+	IVF vs. fertile control	*0.0086/0.043*	*1.57*	*1.13–2.19*	**↑**	S3
2DL5 gr.2/C2+	IVF vs. fertile control	*0.002/0.01*	*1.76*	*1.23–2.53*	**↑**	S3
2DL5 gr.2/C1C2	IVF vs. fertile control	*0.0047/0.024*	*1.78*	*1.19–2.67*	**↑**	S3
2DS2/C1+	IVF vs. fertile control	*0.0034/0.017*	*1.54*	*1.16–2.06*	**↑**	S3
2DS2/C2+	IVF vs. fertile control	*0.0037/0.019*	*1.58*	*1.16–2.14*	**↑**	S3
2DS2/C1C2	IVF vs. fertile control	*0.0045/0.023*	*1.61*	*1.16–2.24*	**↑**	S3
2DS3/C1+	IVF vs. fertile control	*0.0065/0.033*	*1.60*	*1.14–2.23*	**↑**	S3
2DS3/C2+	IVF vs. fertile control	*0.0013/0.0065*	*1.82*	*1.26–2.63*	**↑**	S3
2DS3/C1C2	IVF vs. fertile control	*0.0043/0.022*	*1.81*	*1.20–2.72*	**↑**	S3
AA	IVF vs. fertile control	0.041/ns	0.71	0.52–0.98	↓	2
AA/C2+	IVF vs. fertile control	0.028/ns	0.66	0.45–0.95	↓	S4
AA/C1C2	IVF vs. fertile control	0.034/ns	0.62	0.41–0.96	↓	S4
Bx	IVF vs. fertile control	0.041/ns	1.40	1.02–1.93	↑	2
Bx/C1+	IVF vs. fertile control	0.058	1.35	1.00–1.81	↑	S4
Cen AA	IVF vs. fertile control	*0.0076/0.03*	*0.67*	*0.50–0.89*	**↓**	2
Cen AA/C2+	IVF vs. fertile control	*0.0058/0.029*	*0.64*	*0.47–0.88*	**↓**	S4
Cen AA/C1C2	IVF vs. fertile control	0.012/ns	0.63	0.44–0.89	↓	S4
Cen AA/Tel AA	IVF vs. fertile control	0.041/ns	0.71	0.52–0.98	↓	2
Cen AA/Tel AA/C2+	IVF vs. fertile control	0.028/ns	0.66	0.45–0.95	↓	S4
Cen AA/Tel AA/C1C2	IVF vs. fertile control	0.034/ns	0.62	0.41–0.96	↓	S4
Cen AB+Cen BB	IVF vs. fertile control	*0.0076/0.03*	*1.5*	*1.12–2.00*	**↑**	2
Cen AB/Tel BB	IVF vs. fertile control	0.054/ns	2.66	0.98–7.25	↑	2
Cen AB/C2+	IVF vs. fertile control	0.05/ns	1.38	1.00–1.91	↑	S4
Cen BB/C1+	IVF vs. fertile control	0.033/ns	1.74	1.06–2.85	↑	S4
Cen AB/Tel BB/C2+	IVF vs. fertile control	*0.01/0.05*	*5.56*	*1.26–24.51*	**↑**	S4
Cen AB/Tel BB/C1C2	IVF vs. fertile control	0.05/ns	4.42	0.98–19.90	↑	S4
Cen BB/Tel AA/C1+	IVF vs. fertile control	0.02/ns	2.26	1.12–4.54	↑	S4
Cen BB/Tel AA/C1C2	IVF vs. fertile control	0.04/ns	2.45	1.04–5.77	↑	S4
2DL3	Moderate OS vs. normozoospermia	0.043/ns	2.47	1.00–6.09	↑	S5
2DL5 gr.1	Moderate OS vs. normozoospermia	0.023/ns	1.87	1.09–3.19	↑	S5
2DS5	Moderate OS vs. normozoospermia	0.044/ns	1.78	1.04–3.04	↑	S5
Cen BB	Moderate OS vs. normozoospermia	0.043/ns	0.40	0.16–0.99	↓	S6
Cen AB	Asthenospermic men vs. normospermic men	0.046/ns	1.61	1.01–2.56	↑	S8
Cen AB	Teratospermic men vs. normospermic men	0.015/ns	2.82	1.21–6.55	↑	S8

↑ susceptibility, ↓ protection

The most striking associations are in italics

*NS* not significant after Bonferroni correction

Based on the results of our research, we can conclude that more men reporting for IVF are carriers of genes for the activating receptors KIR2DS2, KIR2DS3, and Cen AB + BB genotypes than men who have children after natural fertilization. In contrast, AA genotype carriers were more frequent in the fertile group. This effect was even maintained in carriers of the combination of AA genotype and the HLA-C2 allotype. The worst KIR-HLA-C combination for male infertility seems to be Cen AB/Tel BB/C2+ (OR = 5.56), where *KIR2DS1*, *KIR2DS5*, and *KIR3DS1* were also included by definition. And indeed, we observed men with moderate oligozoospermia possessed a higher frequency of *KIR2DS5* gene in comparison with normospermic men. Moreover, asthenospermic and teratospermic men were more frequent carriers of Cen AB than normospermic (Table 4). An explanation of our results may be the theory that under certain conditions in the testicles, vas deferens, epididymis, during inflammatory reaction, cytotoxic stimulation of NK cells, and some T lymphocytes by KIR activating receptors may occur. This can happen during urogenital infections which can often be asymptomatic. Action of pathogens themselves and activated immune processes can result in anatomical occlusions, impact the availability of diverse seminal components, impair normal gland function, and disrupt sperm viability [24–26]. Bacteriospermias in general, may reduce semen quality by genital tract dysfunction, impaired spermatogenesis [27, 28]. Also, viruses such as HIV, HPV, and HBV can be internalized by spermatozoa causing DNA damage and altering sperm number, motility, and morphology. In case of HBV, it can also induce the production of antisperm antibodies [26, 29]. Moreover, pathogen-activated immune cells in addition to destroying the pathogen can also destroy own tissues, in this case sperm or tissues involved in the proper development, differentiation, and storage of sperm. Cross-presentation of similar own and pathogen-derived peptides by HLA on own tissues can lead to autoimmunity. Sim et al. showed that presentation of endogenous peptides or HIV Gag peptides by HLA-C can promote KIR cross-reactive binding [30]. Canonical and cross-reactive binding of KIR to HLA-C and the contribution of peptides to the interaction may vary among different KIR–HLA-C combinations, e.g., KIR2DL2 and KIR2DL3 cross-reactive binding with HLA-C C2 has been shown to vary among C2 allotypes [31]. Moreover, allelic polymorphisms even at sites distal to the ligand-binding site of KIR2DL2/3 diversified receptor’s interactions with HLA-C [31]. Another study reported by the group of Sim et al. showed a highly conserved peptide sequence motif for HLA-C*05:01-restricted activation of human KIR2DS4+ NK cells in bacterial recombinase A [30]. NK cells positive for KIR2DS4 were stimulated by this epitope from different human pathogens, such as Helicobacter, Chlamydia, Brucella, and Campylobacter. They predict that over 1000 bacterial species could activate NK cells through KIR2DS4, and propose that human NK cells also contribute to immune defense against bacteria through recognition of a conserved recombinase A epitope presented by HLA-C*05:01. Moreover, Naiyer et al. detected the peptide LNPSVAATL from the hepatitis C virus (HCV) helicase which binds to HLA-C*0102, leading to NK cell activation through engagement of KIR2DS2, and inhibits HCV replication in the context of HLA-C*0102 [32]. During viral infections, T cells are activated in a T cell receptor–independent and cytokine-dependent manner referred to as “bystander activation.” Cytokines, such as type I interferons, interleukin-15, and interleukin-18, are the most important factors that induce bystander activation of T cells. Bystander T cells lack specificity for the pathogen, but can nevertheless impact the course of the immune response to the infection. For example, bystander-activated CD8+ T cells can participate in protective immunity by secreting cytokines, such as interferon-γ. They also mediate host injury by exerting cytotoxicity that is facilitated by natural killer cell-activating receptors, such as NKG2D, and cytolytic molecules, such as granzyme B [33]. We assume that also CD8+ T cells with activating KIR receptors may participate in the destruction of sperm and tissues of the testis, epididymis, and vas deferens. However, this theory requires confirmation.

Considering a role of HLA in the impairment of semen parameters, the study by Marques’ et al. should be noticed [34]. They reported HLA-C*03:03 and HLA-C*06:02 as risk alleles for oligozoospermia and semen hyperviscosity, respectively. Also, HLA-DQB1*03:01 was identified as an oligozoospermia risk allele, whereas HLA-DQB1*03:02 was classified as a protective allele for both oligozoospermia and semen hyperviscosity. Moreover, HLA-A*11:01 was also associated with male infertility. This allele and some alleles belonging to HLA-C C1 allotypes C*01, C*03, C*07, and C*08 were described as ligands for KIR2DS2 [35, 36], whereas HLA-C*03:03 was reported as a ligand for KIR2DL2 and HLA-C*06:02 as a ligand for KIR2DL1 [37]. Therefore, it is possible that upon KIR-HLA interaction, activation of NK cells and some T lymphocytes may occur.

However, HLA class I expression on spermatozoa has been the subject of debate for many years, and the results of the studies are controversial. Some studies indicate both class I and class II molecule HLA expression by spermatozoa [38–40]. However, expression of HLA class I molecules was not detected in all sperm samples of both fertile and infertile men [39]. On the other hand, the class II HLA antigen positivity in the fertile group was significantly lower than in the infertile group. It is worthy to notice that in majority of males positive for class II HLA were detected also varicocele [40]. In addition, class I HLA transcripts were detectable in human spermatogenic cells, such as primary or secondary spermatocytes and spermatids, during spermatogenesis, whereas mature sperm cells do not express detectable amounts of HLA molecules on their cell surface or HLA antigen expression density is actually lower in mature sperm cells [41]. Low levels of expression or a lack of HLA antigens in mature spermatozoa may have biological significance and may contribute to immunological privilege observed in the testis [42] and might protect male gametes from destruction by natural killer cells and T lymphocytes expressing KIRs, either in the male or female reproductive tract. However, as was mentioned earlier, under certain conditions (e.g., infections or hormonal status), this expression could be changed. Moreover, HLA molecules exist in semen in soluble form in seminal plasma and in membrane form on the surface of cells, such as epithelia and leukocytes [43, 44]. That is why their participation should also be discussed.

A limitation of this work is that we did not have the results of sperm parameters from fertile men in the control group. This was for the simple reason of not having to donate sperm because their female partners had no problems conceiving. We could only compare the semen parameters of the men who joined IVF programs. Unfortunately, full data on semen parameters was available for a smaller number of IVF men, which is why some significant results could not withstand correction for multiple comparisons.

Finally, we would also like to emphasize that while maternal KIR AA and fetal/embryo HLA-C2 were associated with reproductive failures [45], our study indicates that in men this genotype and interaction with HLA-C2 may be beneficial to their fertility.

## Conclusions

1. Fertile men differ in profile of *KIR* genes from men participating in IVF.
2. Potential interactions between activating KIRs in Cen AB and BB carriers and HLA-C allotypes may influence male infertility, whereas KIRs in CenAA carriers seems to protect.

## Electronic supplementary material

ESM.1 (DOCX 118 kb)

ESM.2 (DOCX 13 kb)

ESM.3 (DOCX 19 kb)

ESM.4 (DOCX 20 kb)

ESM.5 (DOCX 20 kb)

ESM.6 (DOCX 18 kb)

ESM.7 (DOCX 16 kb)

ESM.8 (DOCX 21 kb)

ESM.9 (DOCX 18 kb)
