# Supplementary material for: KIR and HLA-C genes in male infertility
Source: J Assist Reprod Genet. 2020 May 20;37(8):2007–17. doi: 10.1007/s10815-020-01814-6 (PMC7467998; doi:10.1007/s10815-020-01814-6)
Supplement: Supplementary file 1 — (DOCX 118 kb) [file 10815_2020_1814_MOESM1_ESM.docx]

**Supplementary Fig1.** KIR genotyping

a.


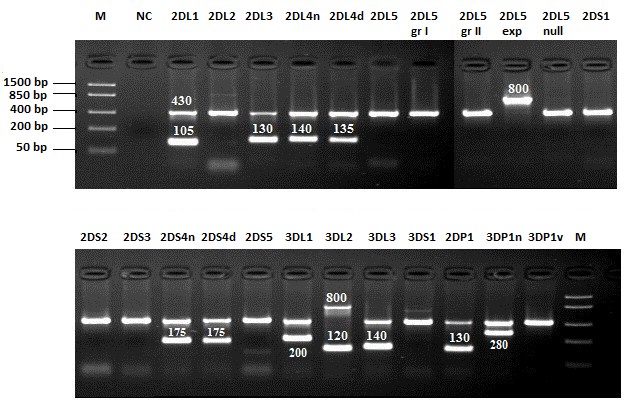


This individual is positive for genes: KIR2DL1, KIR2DL3, KIR2DL4n, KIR2DL4d, KIR2DS4n, KIR2DS4d, KIR3DL1, KIR3DL2, KIR3DL3, KIR2DP1, KIR3DP1n

b.

| **Reagent** | **Volume [μl]** |
| --- | --- |
| Destilled water | 168 |
| Ready PCR | 84 |
| Taq-Polymerase (5 U/μl) | 2,2 |
| DNA (25-100 ng/μl) | 26 |
| Total volume | 280,2 |

PCR reaction:

| Initial denaturation | 96°C | 2 min |  |
| --- | --- | --- | --- |
| Denaturation | 96°C | 15 s. | 10x |
| Annealing | 65°C | 60 s. |  |
| Denaturation | 96°C | 15 s. | 20x |
| Annealing | 61°C | 50 s. |  |
| Extension | 72°C | 30 s. |  |
| ∞ | 4°C |  |  |
