# Supplementary material for: KIR and HLA-C genes in male infertility
Source: J Assist Reprod Genet. 2020 May 20;37(8):2007–17. doi: 10.1007/s10815-020-01814-6 (PMC7467998; doi:10.1007/s10815-020-01814-6)
Supplement: Supplementary file 2 — (DOCX 13 kb) [file 10815_2020_1814_MOESM2_ESM.docx]

**Supplementary Table 1**. Allele specificity

| **KIR Gene Type** | **Allele Specificity** |
| --- | --- |
| 2DL1 | *001-022, 024-026N |
| 2DL2 | *001-003, 005, 007-010, 012 / 2DP1*012 |
| 2DL3 | *001-003, (004), 005-009, 011-017, 019-024 / 2DS4*013 |
| 2DL4 norm | *0001-006, 010, 012, 014-016, 018, 021-026 |
| 2DL4 deleted | *007-009, 011, 013, 017, 019, 020, 027 |
| 2DL5 all | A*all (001, 005, 012, 014, 015), B*all (002-004, 006-011, 013, 016-018) |
| 2DL5 (group 1) | A*001, 012, 014, 015,  B*003, 004, 006-008, 011, 013, 018 |
| 2DL5 (group 2) | A*005, B*002, 009, 010, 016, 017 |
| 2DL5 expressed | A*001, 005:01:01, 005:01:03, 005:01:04, 012,  (014, 015) / 3DP1*004,  (002, 011-014) |
| 2DL5 null | B*002, 004, 006:01, 006:03, 007-011, 013 (0070102, 00803, 01303, 016-018) / 3DP1*001, 007, 009:01, (011-014) |
| 2DS1 | *002, 003, 005, 006 |
| 2DS2 | *001-008 |
| 2DS3 | *001-007 |
| 2DS4 norm | *001, 011, 014, 015 |
| 2DS4 (del-22bp) | *003-010, 012, 013 |
| 2DS5 | *001-012 |
| 3DL1 | *001-008, 015-026, (028), 029-041, 043-056, 059-076 / 3DS1*014 |
| 3DL2 | *001-017, 019-063 |
| 3DL3 | *001-057 |
| 3DS1 | *010-058 |
| 2DP1 | *001-014 |
| 3DP1 norm | *003, 005, 006, 008, 010, 013, 014 |
| 3DP1 variant | *001, 002, 004, 007, 009, 011, 012 |
