# Supplementary material for: KIR and HLA-C genes in male infertility
Source: J Assist Reprod Genet. 2020 May 20;37(8):2007–17. doi: 10.1007/s10815-020-01814-6 (PMC7467998; doi:10.1007/s10815-020-01814-6)
Supplement: Supplementary file 3 — (DOCX 19 kb) [file 10815_2020_1814_MOESM3_ESM.docx]

**Supplementary Table 2**. *KIR* gene frequencies in fertile men and men whose female partners had RSA.

|  | | | | | |
| --- | --- | --- | --- | --- | --- |
| **KIR/HLA-C** | **Fertile control**  **N = 321** | **RSA men**  **N = 298** | **RSA men vs. Fertile control** | | |
|  |  |  | ***P*/*P*_corr._** | **OR** | **95%CI** |
| **KIR** |  |  |  |  |  |
| **2DL1** | 311 (97.19) | 288 (96.64) | 1.00 | 0.93 | 0.38-2.26 |
| **2DL2** | 158 (49.22) | 168 (56.38) | 0.077 | 1.33 | 0.97-1.83 |
| **2DL3** | 290 (90.34) | 262 (87.92) | 0.37 | 0.78 | 0.47-1.29 |
| **2DL5 all** | 145 (45.17) | 155 (52.01) | 0.09 | 1.32 | 0.96-1.81 |
| **2DS1** | 117 (36.45) | 120 (40.27) | 0.36 | 1.18 | 0.85-1.63 |
| **2DS2** | 159 (49.53) | 170 (57.05) | 0.06 | 1.35 | 0.99-1.86 |
| **2DS3** | 82 (25.55) | 100 (33.56) | **0.03/**ns | **1.47** | **1.04-2.08** |
| **2DS4 norm** | 105 (32.71) | 102 (34.23) | 0.73 | 1.07 | 0.77-1.50 |
| **2DS4 del** | 271 (84.42) | 258 (86.58) | 0.49 | 1.19 | 0.76-1.87 |
| **2DS5** | 77 (23.99) | 75 (25.17) | 0.78 | 1.07 | 0.74-1.54 |
| **3DL1** | 303 (94.39) | 283 (94.97) | 0.86 | 1.12 | 0.55-2.27 |
| **3DL2** | 321 (100.0) | 298 (100) | - | - | - |
| **3DL3** | 321 (100.0) | 298 (100) | - | - | - |
| **3DS1** | 107 (33.33) | 111 (37.25) | 0.31 | 1.19 | 0.85-1.65 |
| **HLA-C** |  |  |  |  |  |
| **C1C1** | 111 (34.58) | 111 (37.25) | 0.50 | 1.12 | 0.81-1.56 |
| **C1C2** | 160 (49.84) | 149 (50) | 1.00 | 1.01 | 0.73-1.38 |
| **C2C2** | 50 (15.58) | 38 (12.75) | 0.36 | 0.79 | 0.50-1.25 |
| **C1+** | 382 (59.50) | 371 (62.25) | 0.35 | 1.12 | 0.89-1.41 |
| **C2+** | 260 (40.50) | 225 (37.75) | 0.35 | 0.89 | 0.71-1.12 |
| **KIR/HLA-C** |  |  |  |  |  |
| **2DL1/C1+** | 265 (82.55) | 251 (84.23) | 0.59 | 1.13 | 0.74-1.73 |
| **2DL1/C2+** | 202 (62.93) | 181 (60.74) | 0.62 | 0.91 | 0.66-1.26 |
| **2DL1/C1C1** | 109 (33.96) | 107 (35.91) | 0.61 | 1.09 | 0.78-1.52 |
| **2DL1/C1C2** | 156 (48.60) | 144 (48.32) | 1.00 | 0.99 | 0.72-1.36 |
| **2DL1/C2C2** | 46 (14.33) | 37 (12.42) | 0.56 | 0.85 | 0.53-1.35 |
| **2DL2/C1+** | 135 (42.06) | 148 (49.66) | 0.06 | 1.36 | 0.99-1.87 |
| **2DL2/C2+** | 95 (29.60) | 100 (33.56) | 0.30 | 1.20 | 0.86-1.69 |
| **2DL2/C1C1** | 63 (19.63) | 68 (22.82) | 0.38 | 1.21 | 0.82-1.78 |
| **2DL2/C1C2** | 72 (22.43) | 80 (26.85) | 0.22 | 1.27 | 0.88-1.83 |
| **2DL2/C2C2** | 23 (7.17) | 20 (6.71) | 0.88 | 0.93 | 0.50-1.74 |
| **2DL3/C1+** | 246 (76.64) | 227 (76.17) | 0.92 | 0.97 | 0.67-1.41 |
| **2DL3/C2+** | 188 (58.57) | 166 (55.7) | 0.52 | 0.89 | 0.65-1.22 |
| **2DL3/C1C1** | 102 (31.78) | 96 (32.21) | 0.93 | 1.02 | 0.73-1.43 |
| **2DL3/C1C2** | 144 (44.86) | 131 (43.96) | 0.87 | 0.96 | 0.70-1.32 |
| **2DL3/C2C2** | 44 (13.71) | 35 (11.74) | 0.47 | 0.84 | 0.52-1.35 |
| **2DL5/C1+** | 127 (39.56) | 139 (46.64) | 0.09 | 1.34 | 0.97-1.84 |
| **2DL5/C2+** | 89 (27.73) | 94 (31.54) | 0.33 | 1.20 | 0.85-1.70 |
| **2DL5/C1C1** | 56 (17.45) | 61 (20.47) | 0.36 | 1.22 | 0.81-1.82 |
| **2DL5/C1C2** | 71 (22.12) | 78 (26.17) | 0.26 | 1.25 | 0.86-1.81 |
| **2DL5/C2C2** | 18 (5.61) | 16 (5.37) | 1.00 | 0.96 | 0.48-1.91 |
| **2DS1/C1+** | 106 (33.02) | 108 (36.24) | 0.45 | 1.15 | 0.83-1.61 |
| **2DS1/C2+** | 74 (23.05) | 74 (24.83) | 0.64 | 1.10 | 0.76-1.60 |
| **2DS1/C1C1** | 43 (13.40) | 46 (15.44) | 0.49 | 1.18 | 0.75-1.85 |
| **2DS1/C1C2** | 63 (19.63) | 74 (24.83) | 0.12 | 1.35 | 0.92-1.98 |
| **2DS1/C2C2** | 11 (3.43) | 12 (4.03) | 0.83 | 1.18 | 0.51-2.72 |
| **2DS2/C1+** | 137 (42.68) | 151 (50.67) | **0.05/**ns | **1.38** | **1.01-1.89** |
| **2DS2/C2+** | 96 (29.91) | 102 (34.23) | 0.26 | 1.22 | 0.87-1.71 |
| **2DS2/C1C1** | 63 (19.63) | 68 (22.82) | 0.38 | 1.21 | 0.82-1.78 |
| **2DS2/C1C2** | 74 (23.05) | 83 (27.85) | 0.20 | 1.29 | 0.90-1.85 |
| **2DS2/C2C2** | 22 (6.85) | 19 (6.38) | 0.87 | 0.93 | 0.49-1.75 |
| **2DS3/C1+** | 70 (21.81) | 89 (29.87) | **0.03/**ns | **1.53** | **1.06-2.20** |
| **2DS3/C2+** | 51 (15.89) | 61 (20.47) | 0.14 | 1.36 | 0.90-2.06 |
| **2DS3/C1C1** | 31 (9.66) | 39 (13.09) | 0.20 | 1.41 | 0.85-2.32 |
| **2DS3/C1C2** | 39 (12.15) | 50 (16.78) | 0.11 | 1.46 | 0.93-2.29 |
| **2DS3/C2C2** | 12 (3.74) | 11 (3.69) | 1.00 | 0.99 | 0.43-2.27 |
| **2DS4 norm/C1+** | 81 (25.23) | 88 (29.53) | 0.24 | 1.24 | 0.87-1.77 |
| **2DS4 norm/C2+** | 74 (23.05) | 68 (22.82) | 1.00 | 0.99 | 0.68-1.44 |
| **2DS4 norm/C1C1** | 31 (9.66) | 34 (11.41) | 0.51 | 1.21 | 0.72-2.02 |
| **2DS4 norm/C1C2** | 50 (15.58) | 68 (22.82) | **0.02/**ns | **1.60** | **1.07-2.40** |
| **2DS4 norm/C2C2** | 24 (7.48) | 14 (4.7) | 0.18 | 0.61 | 0.31-1.20 |
| **2DS4 del/C1+** | 229 (71.34) | 224 (75.17) | 0.32 | 1.22 | 0.85-1.74 |
| **2DS4 del/C2+** | 174 (54.21) | 157 (52.68) | 0.75 | 0.94 | 0.69-1.29 |
| **2DS4 del/C1C1** | 97 (30.22) | 101 (33.89) | 0.34 | 1.18 | 0.84-1.66 |
| **2DS4 del/C1C2** | 132 (41.12) | 123 (41.28) | 1.00 | 1.01 | 0.73-1.39 |
| **2DS4 del/C2C2** | 42 (13.08) | 34 (11.41) | 0.54 | 0.86 | 0.53-1.39 |
| **2DS5/C1+** | 70 (21.81) | 69 (23.15) | 0.70 | 1.08 | 0.74-1.58 |
| **2DS5/C2+** | 46 (14.33) | 47 (15.77) | 0.65 | 1.12 | 0.72-1.74 |
| **2DS5/C1C1** | 31 (9.66) | 28 (9.4) | 1.00 | 0.97 | 0.57-1.66 |
| **2DS5/C1C2** | 39 (12.15) | 41 (13.76) | 0.63 | 1.15 | 0.72-1.85 |
| **2DS5/C2C2** | 7 (2.18) | 6 (2.01) | 1.00 | 0.92 | 0.31-2.78 |

Values in bold indicate signiﬁcant differences. Values in parentheses are in percentages. IVF, *in vitro* fertilization; *P*, probability; *P*_corr_., *P* x 14 tested *KIR* variants and *P* x 5 in particular *KIR-HLA-C* combination – Bonferroni correction for multiple comparisons; OR, odds ratio; 95% CI, confidence interval from two-sided Fisher’s exact test; ns, not significant
