# Supplementary material for: KIR and HLA-C genes in male infertility
Source: J Assist Reprod Genet. 2020 May 20;37(8):2007–17. doi: 10.1007/s10815-020-01814-6 (PMC7467998; doi:10.1007/s10815-020-01814-6)
Supplement: Supplementary file 4 — (DOCX 20 kb) [file 10815_2020_1814_MOESM4_ESM.docx]

**Supplementary Table 3**. *KIR* gene frequencies or/and KIR-HLA-C combinations in fertile men and men who participated in IVF

|  | | | | | |
| --- | --- | --- | --- | --- | --- |
| **KIR/HLA-C** | **Fertile control**  **N = 321** | **IVF men**  **N = 445** | **IVF men vs. Fertile control** | | |
|  |  |  | ***P*/*P*_corr._** | **OR** | **95%CI** |
| **HLA-C** |  |  |  |  |  |
| **C1C1** | 111 (34.58) | 157 (35.28) | 0.88 | 1.03 | 0.76-1.39 |
| **C1C2** | 160 (49.84) | 224 (50.34) | 0.94 | 1.02 | 0.77-1.36 |
| **C2C2** | 50 (15.58) | 64 (14.38) | 0.68 | 0.91 | 0.61-1.36 |
| **C1+** | 382 (59.50) | 538 (60.45) | 0.71 | 1.04 | 0.85-1.28 |
| **C2+** | 260 (40.50) | 352 (39.55) | 0.71 | 0.96 | 0.78-1.18 |
| **2DL1/C1+** | 265 (82.55) | 360 (80.90) | 0.57 | 0.90 | 0.62-1.30 |
| **2DL1/C2+** | 202 (62.93) | 274 (61.57) | 0.71 | 0.94 | 0.70-1.27 |
| **2DL1/C1C1** | 109 (33.96) | 149 (33.48) | 0.94 | 0.98 | 0.72-1.33 |
| **2DL1/C1C2** | 156 (48.60) | 211 (47.42) | 0.77 | 0.95 | 0.72-1.27 |
| **2DL1/C2C2** | 46 (14.33) | 62 (13.93) | 0.92 | 0.97 | 0.64-1.46 |
| **2DL2/C1+** | 135 (42.06) | 237 (53.26) | **0.0026/0.013** | **1.57** | **1.18-2.10** |
| **2DL2/C2+** | 95 (29.60) | 179 (40.22) | **0.0029/0.015** | **1.60** | **1.18-2.17** |
| **2DL2/C1C1** | 63 (19.63) | 93 (20.90) | 0.72 | 1.08 | 0.76-1.55 |
| **2DL2/C1C2** | 72 (22.43) | 144 (32.36) | **0.0026/0.013** | **1.65** | **1.19-2.30** |
| **2DL2/C2C2** | 23 (7.17) | 34 (7.64) | 0.89 | 1.07 | 0.62-1.86 |
| **2DL3/C1+** | 246 (76.64) | 324 (72.81) | 0.24 | 0.82 | 0.59-1.14 |
| **2DL3/C2+** | 188 (58.57) | 249 (55.96) | 0.51 | 0.90 | 0.67-1.20 |
| **2DL3/C1C1** | 102 (31.78) | 137 (30.79) | 0.81 | 0.96 | 0.70-1.30 |
| **2DL3/C1C2** | 144 (44.86) | 187 (42.02) | 0.46 | 0.89 | 0.67-1.19 |
| **2DL3/C2C2** | 44 (13.71) | 61 (13.71) | 1.00 | 1.00 | 0.66-1.52 |
| **2DL5/C1+** | 127 (39.56) | 200 (44.94) | 0.14 | 1.25 | 0.93-1.67 |
| **2DL5/C2+** | 89 (27.73) | 153 (34.38) | 0.059 | 1.37 | 1.00-1.87 |
| **2DL5/C1C1** | 56 (17.45) | 80 (17.98) | 0.92 | 1.04 | 0.71-1.51 |
| **2DL5/C1C2** | 71 (22.12) | 120 (26.97) | 0.13 | 1.30 | 0.93-1.82 |
| **2DL5/C2C2** | 18 (5.61) | 33 (7.42) | 0.38 | 1.35 | 0.74-2.44 |
| **2DL5 gr.1/C1+** | 70 (21.81) | 105 (23.60) | 0.60 | 1.11 | 0.79-1.56 |
| **2DL5 gr.1/C2+** | 45 (14.02) | 74 (16.63) | 0.36 | 1.22 | 0.82-1.83 |
| **2DL5 gr.1/C1C1** | 32 (9.97) | 46 (10.34) | 0.90 | 1.04 | 0.65-1.68 |
| **2DL5 gr.1/C1C2** | 38 (11.84) | 59 (13.26) | 0.58 | 1.14 | 0.74-1.76 |
| **2DL5 gr.1/C2C2** | 7 (2.18) | 15 (3.37) | 0.39 | 1.57 | 0.63-3.88 |
| **2DL5 gr.2/C1+** | 72 (22.43) | 139 (31.24) | **0.0086/0.043** | **1.57** | **1.13-2.19** |
| **2DL5 gr.2/C2+** | 53 (16.51) | 115 (25.84) | **0.002/0.01** | **1.76** | **1.23-2.53** |
| **2DL5 gr.2/C1C1** | 32 (9.97) | 49 (11.01) | 0.72 | 1.12 | 0.70-1.79 |
| **2DL5 gr.2/C1C2** | 40 (12.46) | 90 (20.22) | **0.0047/0.024** | **1.78** | **1.19-2.67** |
| **2DL5 gr.2/C2C2** | 13 (4.05) | 25 (5.62) | 0.40 | 1.41 | 0.71-2.80 |
| **2DL5 exp./C1+** | 94 (29.28) | 144 (32.36) | 0.38 | 1.16 | 0.85-1.58 |
| **2DL5 exp./C2+** | 62 (19.31) | 105 (23.60) | 0.18 | 1.29 | 0.91-1.84 |
| **2DL5 exp./C1C1** | 41 (12.77) | 60 (13.48) | 0.83 | 1.06 | 0.70-1.63 |
| **2DL5 exp./C1C2** | 53 (16.51) | 84 (18.88) | 0.44 | 1.18 | 0.81-1.72 |
| **2DL5 exp./C2C2** | 9 (2.80) | 21 (4.72) | 0.19 | 1.72 | 0.78-3.80 |
| **2DS1/C1+** | 106 (33.02) | 149 (33.48) | 0.94 | 1.02 | 0.75-1.39 |
| **2DS1/C2+** | 74 (23.05) | 108 (24.27) | 0.73 | 1.07 | 0.76-1.50 |
| **2DS1/C1C1** | 43 (13.40) | 63 (14.16) | 0.83 | 1.07 | 0.70-1.62 |
| **2DS1/C1C2** | 63 (19.63) | 86 (19.33) | 0.93 | 0.98 | 0.68-1.41 |
| **2DS1/C2C2** | 11 (3.43) | 22 (4.94) | 0.37 | 1.47 | 0.70-3.07 |
| **2DS2/C1+** | 137 (42.68) | 238 (53.48) | **0.0034/0.017** | **1.54** | **1.16-2.06** |
| **2DS2/C2+** | 96 (29.91) | 179 (40.22) | **0.0037/0.019** | **1.58** | **1.16-2.14** |
| **2DS2/C1C1** | 63 (19.63) | 93 (20.90) | 0.72 | 1.08 | 0.76-1.55 |
| **2DS2/C1C2** | 74 (23.05) | 145 (32.58) | **0.0045/0.023** | **1.61** | **1.16-2.24** |
| **2DS2/C2C2** | 22 (6.85) | 33 (7.42) | 0.89 | 1.09 | 0.62-1.91 |
| **2DS3/C1+** | 70 (21.81) | 137 (30.79) | **0.0065/0.033** | **1.60** | **1.14-2.23** |
| **2DS3/C2+** | 51 (15.89) | 114 (25.62) | **0.0013/0.0065** | **1.82** | **1.26-2.63** |
| **2DS3/C1C1** | 31 (9.66) | 48 (10.79) | 0.63 | 1.13 | 0.70-1.82 |
| **2DS3/C1C2** | 39 (12.15) | 89 (20.00) | **0.0043/0.022** | **1.81** | **1.20-2.72** |
| **2DS3/C2C2** | 12 (3.74) | 25 (5.62) | 0.31 | 1.53 | 0.76-3.10 |
| **2DS4 norm/C1+** | 81 (25.23) | 134 (30.11) | 0.14 | 1.28 | 0.92-1.76 |
| **2DS4 norm/C2+** | 74 (23.05) | 102 (22.92) | 1.00 | 0.99 | 0.71-1.40 |
| **2DS4 norm/C1C1** | 31 (9.66) | 57 (12.81) | 0.21 | 1.37 | 0.86-2.18 |
| **2DS4 norm/C1C2** | 50 (15.58) | 77 (17.30) | 0.56 | 1.13 | 0.77-1.67 |
| **2DS4 norm/C2C2** | 24 (7.48) | 24 (5.39) | 0.29 | 0.71 | 0.39-1.27 |
| **2DS4 del/C1+** | 229 (71.34) | 309 (69.44) | 0.58 | 0.91 | 0.67-1.25 |
| **2DS4 del/C2+** | 174 (54.21) | 236 (53.03) | 0.77 | 0.95 | 0.72-1.27 |
| **2DS4 del/C1C1** | 97 (30.22) | 130 (29.21) | 0.81 | 0.95 | 0.70-1.30 |
| **2DS4 del/C1C2** | 132 (41.12) | 179 (40.22) | 0.82 | 0.96 | 0.72-1.29 |
| **2DS4 del/C2C2** | 42 (13.08) | 56 (12.58) | 0.91 | 0.96 | 0.62-1.47 |
| **2DS5/C1+** | 70 (21.81) | 104 (23.37) | 0.66 | 1.09 | 0.78-1.54 |
| **2DS5/C2+** | 46 (14.33) | 74 (16.63) | 0.42 | 1.19 | 0.80-1.78 |
| **2DS5/C1C1** | 31 (9.66) | 45 (10.11) | 0.90 | 1.05 | 0.65-1.70 |
| **2DS5/C1C2** | 39 (12.15) | 59 (13.26) | 0.66 | 1.11 | 0.72-1.70 |
| **2DS5/C2C2** | 7 (2.18) | 15 (3.37) | 0.39 | 1.57 | 0.63-3.88 |

Values in bold indicate signiﬁcant differences. Values in parentheses are in percentages. IVF, *in vitro* fertilization; *P*, probability; *P*_corr_., *P* x 5 for particular *KIR-HLA-C* gene combination – Bonferroni correction for multiple comparisons; OR, odds ratio; 95% CI, confidence interval from two-sided Fisher’s exact test; ns, not significant
