# Supplementary material for: KIR and HLA-C genes in male infertility
Source: J Assist Reprod Genet. 2020 May 20;37(8):2007–17. doi: 10.1007/s10815-020-01814-6 (PMC7467998; doi:10.1007/s10815-020-01814-6)
Supplement: Supplementary file 5 — (DOCX 20 kb) [file 10815_2020_1814_MOESM5_ESM.docx]

**Supplementary Table 4.**  Centromeric and telomeric *KIR* genotypes with *HLA-C* combinations in fertile men and men who participated in IVF

|  | | | | | |
| --- | --- | --- | --- | --- | --- |
| **KIR/HLA-C combination** | **Fertile control**  **N = 321** | **IVF men**  **N = 445** | **IVF men vs. Fertile control** | | |
|  |  |  | ***P*/*P*_corr._** | **OR** | **95%CI** |
| **AA/C1+** | 82 (25.55) | 88 (19.78) | 0.064 | 0.72 | 0.51-1.07 |
| **AA/C2+** | 69 (21.50) | 68 (15.28) | **0.028/**ns | **0.66** | **0.45-0.95** |
| **AA/C1C1** | 33 (10.28) | 43 (9.66) | 0.81 | 0.93 | 0.58-1.51 |
| **AA/C1C2** | 49 (15.26) | 45 (10.11) | **0.034/**ns | **0.62** | **0.41-0.96** |
| **AA/C2C2** | 20 (6.23) | 23 (5.17) | 0.53 | 0.82 | 0.44-1.52 |
| **Bx/C1+** | 189 (58.88) | 293 (65.84) | 0.058 | 1.35 | 1.00-1.81 |
| **Bx/C2+** | 141 (43.93) | 221 (49.66) | 0.12 | 1.26 | 0.94-1.68 |
| **Bx/C1C1** | 78 (24.30) | 114 (25.62) | 0.74 | 1.07 | 0.77-1.50 |
| **Bx/C1C2** | 111 (34.58) | 179 (40.22) | 0.11 | 1.27 | 0.95-1.72 |
| **Bx/C2C2** | 30 (9.35) | 41 (9.21) | 1.00 | 0.98 | 0.60-1.61 |
| **Cen AA/C1+** | 129 (40.19) | 141 (31.69) | **0.018/**ns | **0.69** | **0.51-0.93** |
| **Cen AA/C2+** | 108 (33.64) | 109 (24.49) | **0.0058/0.029** | **0.64** | **0.47-0.88** |
| **Cen AA/C1C1** | 47 (14.64) | 62 (13.93) | 0.25 | 1.28 | 0.84-1.93 |
| **Cen AA/C1C2** | 82 (25.55) | 79 (17.75) | **0.012/**ns | **0.63** | **0.44-0.89** |
| **Cen AA/C2C2** | 26 (8.10) | 30 (6.74) | 0.49 | 0.82 | 0.48-1.42 |
| **Cen AB/C1+** | 117 (36.45) | 183 (41.12) | 0.20 | 1.22 | 0.91-1.64 |
| **Cen AB/C2+** | 80 (24.92) | 140 (31.46) | **0.05/**ns | **1.38** | **1.00-1.91** |
| **Cen AB/C1C1** | 55 (17.13) | 75 (16.85) | 0.92 | 0.98 | 0.67-1.44 |
| **Cen AB/C1C2** | 62 (19.31) | 108 (24.27) | 0.11 | 1.34 | 0.94-1.90 |
| **Cen AB/C2C2** | 18 (5.61) | 31 (6.97) | 0.55 | 1.26 | 0.69-2.30 |
| **Cen BB/C1+** | 25 (7.79) | 57 (12.81) | **0.033/**ns | **1.74** | **1.06-2.85** |
| **Cen BB/C2+** | 22 (6.85) | 40 (8.99) | 0.35 | 1.34 | 0.78-2.31 |
| **Cen BB/C1C1** | 9 (2.80) | 20 (4.49) | 0.25 | 1.63 | 0.73-3.63 |
| **Cen BB/C1C2** | 16 (4.98) | 37 (8.31) | 0.084 | 1.73 | 0.94-3.17 |
| **Cen BB/C2C2** | 6 (1.87) | 3 (0.67) | 0.18 | 0.36 | 0.09-1.44 |
| **Tel AA/C1+** | 159 (49.53) | 222 (49.89) | 0.94 | 1.01 | 0.76-1.35 |
| **Tel AA/C2+** | 132 (41.12) | 172 (38.65) | 0.50 | 0.90 | 0.67-1.21 |
| **Tel AA/C1C1** | 65 (20.25) | 91 (20.45) | 1.00 | 1.01 | 0.71-1.45 |
| **Tel AA/C1C2** | 94 (29.28) | 131 (29.44) | 1.00 | 1.01 | 0.74-1.38 |
| **Tel AA/C2C2** | 38 (11.84) | 40 (8.99) | 0.22 | 0.74 | 0.46-1.18 |
| **Tel AB/C1+** | 100 (31.15) | 136 (30.56) | 0.87 | 0.97 | 0.71-1.33 |
| **Tel AB/C2+** | 70 (21.81) | 96 (21.57) | 1.00 | 0.99 | 0.70-1.40 |
| **Tel AB/C1C1** | 41 (12.77) | 61 (13.71) | 0.75 | 1.09 | 0.71-1.66 |
| **Tel AB/C1C2** | 59 (18.38) | 75 (16.85) | 0.63 | 0.90 | 0.62-1.31 |
| **Tel AB/C2C2** | 11 (3.43) | 21 (4.72) | 0.47 | 1.40 | 0.66-2.94 |
| **Tel BB/C1+** | 12 (3.74) | 23 (5.17) | 0.39 | 1.40 | 0.69-2.86 |
| **Tel BB/C2+** | 8 (2.49) | 21 (4.72) | 0.13 | 1.94 | 0.85-4.43 |
| **Tel BB/C1C1** | 5 (1.56) | 5 (1.12) | 0.75 | 0.72 | 0.21-2.50 |
| **Tel BB/C1C2** | 7 (2.18) | 18 (4.04) | 0.22 | 1.89 | 0.78-4.58 |
| **Tel BB/C2C2** | 1 (0.31) | 3 (0.67) | 0.64 | 2.17 | 0.22-20.99 |
| **Cen AA/Tel AA/C1+** | 82 (25.55) | 88 (19.78) | 0.06 | 0.72 | 0.51-1.01 |
| **Cen AA/Tel AA/C2+** | 69 (21.50) | 68 (15.28) | **0.028/**ns | **0.66** | **0.45-0.95** |
| **Cen AA/Tel AA/C1C1** | 33 (10.28) | 43 (9.66) | 0.81 | 0.93 | 0.58-1.51 |
| **Cen AA/Tel AA/C1C2** | 49 (15.26) | 45 (10.11) | **0.034/**ns | **0.62** | **0.41-0.96** |
| **Cen AA/Tel AA/C2C2** | 20 (6.23) | 23 (5.17) | 0.53 | 0.82 | 0.44-1.52 |
| **Cen AA/Tel AB/C1+** | 43 (13.40) | 50 (11.24) | 0.37 | 0.82 | 0.53-1.27 |
| **Cen AA/Tel AB/C2+** | 36 (11.21) | 38 (8.54) | 0.22 | 0.74 | 0.46-1.20 |
| **Cen AA/Tel AB/C1C1** | 13 (4.05) | 19 (4.27) | 1.00 | 1.06 | 0.51-2.17 |
| **Cen AA/Tel AB/C1C2** | 30 (9.35) | 31 (6.97) | 0.28 | 0.73 | 0.43-1.23 |
| **Cen AA/Tel AB/C2C2** | 6 (1.87) | 7 (1.57) | 0.78 | 0.84 | 0.28-2.52 |
| **Cen AA/Tel BB/C1+** | 4 (1.25) | 3 (0.67) | 0.46 | 0.54 | 0.12-2.42 |
| **Cen AA/Tel BB/C2+** | 3 (0.93) | 3 (0.67) | 0.70 | 0.72 | 0.14-3.59 |
| **Cen AA/Tel BB/C1C1** | 1 (0.31) | 0 (0.00) | 0.42 | 0.24 | 0.01-5.91 |
| **Cen AA/Tel BB/C1C2** | 3 (0.93) | 3 (0.67) | 0.70 | 0.72 | 0.14-3.59 |
| **Cen AA/Tel BB/C2C2** | 0 (0.00) | 0 (0.00) | - | - | - |
| **Cen AB/Tel AA/C1+** | 66 (20.56) | 101 (22.70) | 0.53 | 1.13 | 0.80-1.61 |
| **Cen AB/Tel AA/C2+** | 52 (16.20) | 79 (17.75) | 0.63 | 1.12 | 0.76-1.64 |
| **Cen AB/Tel AA/C1C1** | 28 (8.72) | 38 (8.54) | 1.00 | 0.98 | 0.59-1.63 |
| **Cen AB/Tel AA/C1C2** | 38 (11.84) | 63 (14.16) | 0.39 | 1.23 | 0.80-1.89 |
| **Cen AB/Tel AA/C2C2** | 14 (4.36) | 15 (3.37) | 0.57 | 0.77 | 0.36-1.61 |
| **Cen AB/Tel AB/C1+** | 46 (14.33) | 66 (14.83) | 0.92 | 1.04 | 0.69-1.56 |
| **Cen AB/Tel AB/C2+** | 26 (8.10) | 46 (10.34) | 0.32 | 1.31 | 0.79-2.17 |
| **Cen AB/Tel AB/C1C1** | 24 (7.48) | 33 (7.42) | 1.00 | 0.97 | 0.56-1.68 |
| **Cen AB/Tel AB/C1C2** | 22 (6.85) | 33 (7.42) | 0.89 | 1.07 | 0.61-1.86 |
| **Cen AB/Tel AB/C2C2** | 4 (1.25) | 13 (2.92) | 0.14 | 2.39 | 0.77-7.39 |
| **Cen AB/Tel BB/C1+** | 5 (1.56) | 16 (3.60) | 0.12 | 2.36 | 0.85-6.50 |
| **Cen AB/Tel BB/C2+** | 2 (0.62) | 15 (3.37) | **0.01/0.05** | **5.56** | **1.26-24.51** |
| **Cen AB/Tel BB/C1C1** | 3 (0.93) | 4 (0.90) | 1.00 | 0.96 | 0.21-4.33 |
| **Cen AB/Tel BB/C1C2** | 2 (0.62) | 12 (2.70) | **0.05/**ns | **4.42** | **0.98-19.90** |
| **Cen AB/Tel BB/C2C2** | 0 (0.00) | 3 (0.67) | 0.27 | 5.09 | 0.26-98.89 |
| **Cen BB/Tel AA/C1+** | 11 (3.43) | 33 (7.42) | **0.02/**ns | **2.26** | **1.12-4.54** |
| **Cen BB/Tel AA/C2+** | 11 (3.43) | 25 (5.62) | 0.17 | 1.68 | 0.81-3.46 |
| **Cen BB/Tel AA/C1C1** | 4 (1.25) | 10 (2.25) | 0.42 | 1.82 | 0.57-5.86 |
| **Cen BB/Tel AA/C1C2** | 7 (2.18) | 23 (5.17) | **0.04/**ns | **2.45** | **1.04-5.77** |
| **Cen BB/Tel AA/C2C2** | 4 (1.25) | 2 (0.45) | 0.24 | 0.36 | 0.07-1.97 |
| **Cen BB/Tel AB/C1+** | 11 (3.43) | 20 (4.49) | 0.58 | 1.33 | 0.63-2.81 |
| **Cen BB/Tel AB/C2+** | 8 (2.49) | 12 (2.70) | 1.00 | 1.08 | 0.44-2.68 |
| **Cen BB/Tel AB/C1C1** | 4 (1.25) | 9 (2.02) | 0.57 | 1.64 | 0.50-5.36 |
| **Cen BB/Tel AB/C1C2** | 7 (2.18) | 11 (2.47) | 1.00 | 1.14 | 0.44-2.97 |
| **Cen BB/Tel AB/C2C2** | 1 (0.31) | 1 (0.22) | 1.00 | 0.72 | 0.04-11.57 |
| **Cen BB/Tel BB/C1+** | 3 (0.93) | 4 (0.90) | 1.00 | 0.96 | 0.21-4.33 |
| **Cen BB/Tel BB/C2+** | 3 (0.93) | 3 (0.67) | 0.70 | 0.72 | 0.14-3.59 |
| **Cen BB/Tel BB/C1C1** | 1 (0.31) | 1 (0.22) | 1.00 | 0.72 | 0.04-11.57 |
| **Cen BB/Tel BB/C1C2** | 2 (0.62) | 3 (0.67) | 1.00 | 1.08 | 0.18-6.52 |
| **Cen BB/Tel BB/C2C2** | 1 (0.31) | 0 (0.00) | 0.42 | 0.24 | 0.01-5.91 |

Values in bold indicate signiﬁcant differences. Values in parentheses are in percentages. IVF, *in vitro* fertilization; *P*, probability; *P*_corr_., *P* x 5 – for particular *KIR* genotype and *HLA-C* combination - Bonferroni correction for multiple comparisons; OR, odds ratio; 95% CI, confidence interval from two-sided Fisher’s exact test; ns, not significant
