# Supplementary material for: KIR and HLA-C genes in male infertility
Source: J Assist Reprod Genet. 2020 May 20;37(8):2007–17. doi: 10.1007/s10815-020-01814-6 (PMC7467998; doi:10.1007/s10815-020-01814-6)
Supplement: Supplementary file 6 — (DOCX 18 kb) [file 10815_2020_1814_MOESM6_ESM.docx]

**Supplementary Table 5**. *KIR* gene frequencies in IVF men stratified according to sperm concentration

| **KIR** | **Normozoospermia**  **N = 234** | **Moderate OS**  **N = 85** | **Severe, very severe OS**  **and AS**  **N = 91** | **Moderate OS**  **vs.**  **Normozoospermia** | | | **Severe, very severe OS and AS**  **vs.**  **Normozoospermia** | | | **Severe, very severe OS and AS**  **vs.**  **Moderate oligozoospermia** | | |
| --- | --- | --- | --- | --- | --- | --- | --- | --- | --- | --- | --- | --- |
|  |  |  |  | ***P*/*P*_corr._** | **OR** | **95%CI** | ***P*/*P*_corr._** | **OR** | **95%CI** | ***P*/*P*_corr._** | **OR** | **95%CI** |
| **2DL1** | 221 (94.44) | 81 (95.29) | 86 (94.51) | 1.00 | 1.19 | 0.38-3.76 | 1.00 | 1.01 | 0.35-2.92 | 1.00 | 0.85 | 0.22-3.28 |
| **2DL2** | 140 (59.83) | 53 (62.35) | 54 (59.34) | 0.70 | 1.11 | 0.67-1.85 | 1.00 | 0.98 | 0.60-1.61 | 0.76 | 0.88 | 0.48-1.62 |
| **2DL3** | 197 (84.19) | 79 (92.94) | 77 (84.62) | **0.043**/ns | **2.47** | **1.00-6.09** | 1.00 | 1.03 | 0.53-2.02 | 0.099 | 0.42 | 0.15-1.14 |
| **2DL4 norm** | 168 (71.79) | 66 (77.65) | 60 (65.93) | 0.32 | 1.37 | 0.76-2.45 | 0.34 | 0.76 | 0.45-1.28 | 0.096 | 0.56 | 0.29-1.09 |
| **2DL4 del** | 177 (75.64) | 67 (78.82) | 73 (80.22) | 0.65 | 1.2 | 0.66-2.18 | 0.46 | 1.31 | 0.72-2.37 | 0.85 | 1.09 | 0.52-2.27 |
| **2DL5 all** | 124 (52.99) | 44 (51.76) | 43 (47.25) | 0.90 | 0.95 | 0.58-1.57 | 0.39 | 0.79 | 0.49-1.29 | 0.65 | 0.83 | 0.46-1.51 |
| **2DL5 gr.1** | 55 (23.50) | 31 (36.47) | 23 (25.27) | **0.023**/ns | **1.87** | **1.09-3.19** | 0.77 | 1.1 | 0.63-1.93 | 0.14 | 0.59 | 0.31-1.13 |
| **2DL5 gr.2** | 92 (39.32) | 26 (30.59) | 29 (31.87) | 0.19 | 0.68 | 0.4-1.16 | 0.25 | 0.72 | 0.43-1.21 | 0.87 | 1.06 | 0.56-2.01 |
| **2DL5 exp** | 86 (36.75) | 33 (38.82) | 33 (36.26) | 0.79 | 1.09 | 0.66-1.82 | 1.00 | 0.98 | 0.59-1.62 | 0.75 | 0.90 | 0.49-1.65 |
| **2DL5 null** | 100 (42.74) | 31 (36.47) | 28 (30.77) | 0.37 | 0.77 | 0.46-1.28 | 0.058 | 0.6 | 0.36-1.00 | 0.43 | 0.77 | 0.41-1.45 |
| **2DS1** | 85 (36.32) | 39 (45.88) | 33 (36.26) | 0.15 | 1.49 | 0.90-2.46 | 1.00 | 1.00 | 0.60-1.65 | 0.22 | 0.67 | 0.37-1.23 |
| **2DS2** | 139 (59.40) | 53 (62.35) | 55 (60.44) | 0.70 | 1.13 | 0.68-1.89 | 0.90 | 1.04 | 0.64-1.71 | 0.88 | 0.92 | 0.5-1.69 |
| **2DS3** | 91 (38.89) | 26 (30.59) | 28 (30.77) | 0.19 | 0.69 | 0.41-1.18 | 0.20 | 0.7 | 0.42-1.17 | 1.00 | 1.01 | 0.53-1.92 |
| **2DS4 norm** | 93 (39.74) | 30 (35.21) | 27 (29.67) | 0.52 | 0.83 | 0.49-1.39 | 0.098 | 0.64 | 0.38-1.08 | 0.52 | 0.77 | 0.41-1.46 |
| **2DS4 del** | 188 (80.34) | 71 (83.53) | 76 (83.52) | 0.63 | 1.24 | 0.64-2.40 | 0.64 | 1.24 | 0.65-2.35 | 1.00 | 1.00 | 0.45-2.22 |
| **2DS5** | 55 (23.50) | 30 (35.21) | 23 (25.27) | **0.044**/ns | **1.78** | **1.04-3.04** | 0.77 | 1.1 | 0.63-1.93 | 0.19 | 0.62 | 0.32-1.19 |
| **3DL1** | 219 (93.59) | 81 (95.29) | 84 (92.31) | 0.79 | 1.39 | 0.45-4.30 | 0.63 | 0.82 | 0.32-2.09 | 0.54 | 0.58 | 0.17-2.10 |
| **3DL2** | 233 (99.57) | 84 (98.82) | 91 (100.0) | 0.46 | 0.36 | 0.02-5.83 | - | - | - | - | - | - |
| **3DL3** | 234 (100.0) | 85 (100.0) | 91 (100.0) | - | - | - | - | - | - | - | - | - |
| **3DS1** | 86 (36.75) | 34 (40.00) | 32 (35.16) | 0.6 | 1.15 | 0.69-1.91 | 0.9 | 0.93 | 0.56-1.55 | 0.54 | 0.81 | 0.44-1.50 |
| **2DP1** | 222 (94.87) | 82 (96.47) | 86 (94.51) | 0.77 | 1.48 | 0.41-5.37 | 1.00 | 0.93 | 0.32-2.72 | 0.72 | 0.63 | 0.15-2.72 |
| **3DP1** | 221 (94.44) | 81 (95.29) | 86 (94.51) | 1.00 | 1.19 | 0.38-3.76 | 1.00 | 1.01 | 0.35-2.92 | 1.00 | 0.85 | 0.22-3.28 |
| **3DP1 var** | 76 (32.48) | 32 (37.65) | 40 (43.96) | 0.42 | 1.26 | 0.75-2.11 | 0.055 | 1.63 | 0.99-2.68 | 0.44 | 1.3 | 0.71-2.38 |

Values in bold indicate signiﬁcant differences. Values in parentheses are in percentages. Normozoospermia (N ≥ 15 mln/mL of sperm cells); OS – oligozoospermia; Moderate OS (5 < N <15 mln/mL); Severe (1-5 mln/mL), very severe OS (N < 1 mln/mL); AS - azoospermia (lack of sperm cells in ejaculate); IVF, *in vitro* fertilization; *P*, probability; *P*_corr_., *P* x 23 tested *KIR* variants – Bonferroni correction for multiple comparisons; OR, odds ratio; 95% CI, confidence interval from two-sided Fisher’s exact test; ns, not significant
