# Supplementary material for: KIR and HLA-C genes in male infertility
Source: J Assist Reprod Genet. 2020 May 20;37(8):2007–17. doi: 10.1007/s10815-020-01814-6 (PMC7467998; doi:10.1007/s10815-020-01814-6)
Supplement: Supplementary file 7 — (DOCX 16 kb) [file 10815_2020_1814_MOESM7_ESM.docx]

| **KIR genotype** | **Normozoospermia**  **N = 234** | **Moderate OS**  **N = 85** | **Severe, very severe OS and AS**  **N = 91** | **Moderate OS vs. Normozoospermia** | | | **Severe, very severe OS and AS vs. Normozoospermia** | | | **Severe, very severe OS and AS vs.**  **Moderate OS** | | |
| --- | --- | --- | --- | --- | --- | --- | --- | --- | --- | --- | --- | --- |
|  |  |  |  | ***P*/*P*_corr._** | **OR** | **95%CI** | ***P*/*P*_corr._** | **OR** | **95%CI** | ***P*/*P*_corr._** | **OR** | **95%CI** |
| AA | 64 (27.35) | 18 (21.18) | 22 (24.18) | 0.31 | 0.71 | 0.39-1.29 | 0.67 | 0.85 | 0.48-1.48 | 0.72 | 1.19 | 0.58-2.41 |
| Bx | 170 (72.65) | 67 (78.82) | 69 (75.82) | 0.31 | 1.40 | 0.77-2.54 | 0.67 | 1.18 | 0.67-2.07 | 0.72 | 0.84 | 0.42-1.71 |
|  |  |  |  |  |  |  |  |  |  |  |  |  |
| Cen AA | 92 (39.32) | 32 (37.65) | 36 (39.56) | 0.90 | 0.93 | 0.56-1.55 | 1.00 | 1.01 | 0.62-1.66 | 0.88 | 1.08 | 0.59-1.99 |
| Cen AB | 105 (44.87) | 47 (55.29) | 41 (45.05) | 0.13 | 1.52 | 0.92-2.50 | 1.00 | 1.01 | 0.62-1.64 | 0.23 | 0.66 | 0.37-1.20 |
| Cen BB | 37 (15.81) | 6 (7.06) | 14 (15.38) | **0.043**/ns | **0.40** | **0.16-0.99** | 1.00 | 0.97 | 0.50-1.89 | 0.099 | 2.39 | 0.87-6.55 |
| Cen AB+Cen BB | 142 (60.68) | 53 (62.35) | 55 (60.44) | 0.90 | 1.07 | 0.64-1.79 | 1.00 | 0.99 | 0.60-1.63 | 0.88 | 0.92 | 0.50-1.69 |
|  |  |  |  |  |  |  |  |  |  |  |  |  |
| Tel AA | 140 (59.83) | 46 (54.12) | 56 (61.54) | 0.37 | 0.79 | 0.48-1.31 | 0.80 | 1.07 | 0.65-1.77 | 0.36 | 1.36 | 0.74-2.47 |
| Tel AB | 80 (34.19) | 35 (41.18) | 30 (32.97) | 0.29 | 1.35 | 0.81-2.24 | 0.90 | 0.95 | 0.57-1.58 | 0.28 | 0.70 | 0.38-1.30 |
| Tel BB | 14 (5.98) | 4 (4.71) | 5 (5.49) | 0.79 | 0.78 | 0.25-2.43 | 1.00 | 0.91 | 0.32-2.61 | 1.00 | 1.18 | 0.31-4.54 |
| Tel AB+Tel BB | 94 (40.17) | 39 (45.88) | 35 (38.46) | 0.37 | 1.26 | 0.77-2.08 | 0.80 | 0.93 | 0.57-1.53 | 0.36 | 0.74 | 0.40-1.34 |
|  |  |  |  |  |  |  |  |  |  |  |  |  |
| Cen AA/Tel AA | 64 (27.35) | 18 (21.18) | 22 (24.18) | 0.31 | 0.71 | 0.39-1.29 | 0.67 | 0.85 | 0.48-1.48 | 0.72 | 1.19 | 0.58-2.41 |
| Cen AA/Tel AB | 27 (11.54) | 13 (15.29) | 13 (14.29) | 0.44 | 1.38 | 0.68-2.83 | 0.57 | 1.28 | 0.63-2.60 | 1.00 | 0.92 | 0.40-2.12 |
| Cen AA/Tel BB | 1 (0.43) | 1 (1.18) | 1 (1.1) | 0.46 | 2.77 | 0.17-44.88 | 0.48 | 2.59 | 0.16-41.86 | 1.00 | 0.93 | 0.06-15.17 |
| Cen AB/Tel AA | 55 (23.50) | 25 (29.41) | 25 (27.47) | 0.31 | 1.36 | 0.78-2.37 | 0.48 | 1.23 | 0.71-2.14 | 0.87 | 0.91 | 0.47-1.75 |
| Cen AB/Tel AB | 40 (17.10) | 19 (22.35) | 12 (13.19) | 0.33 | 1.40 | 0.76-2.58 | 0.50 | 0.74 | 0.37-1.48 | 0.12 | 0.53 | 0.24-1.17 |
| Cen AB/Tel BB | 10 (4.27) | 3 (3.53) | 4 (4.40) | 1.00 | 0.82 | 0.22-3.05 | 1.00 | 1.03 | 0.31-3.37 | 1.00 | 1.26 | 0.27-5.79 |
| Cen BB/Tel AA | 21 (8.97) | 3 (3.53) | 9 (9.90) | 0.15 | 0.37 | 0.11-1.28 | 0.83 | 1.11 | 0.49-2.53 | 0.13 | 3.00 | 0.78-11.48 |
| Cen BB/Tel AB | 13 (5.56) | 3 (3.53) | 5 (5.49) | 0.57 | 0.62 | 0.17-2.24 | 1.00 | 0.99 | 0.34-2.86 | 0.72 | 1.59 | 0.37-6.87 |
| Cen BB/Tel BB | 3 (1.28) | 0 (0.00) | 0 (0.00) | 0.57 | 0.39 | 0.02-7.57 | 0.56 | 0.36 | 0.02-7.07 | - | - | - |

**Supplementary Table 6.** Centromeric and telomeric *KIR* genotypes in IVF men stratified according to sperm concentration

Values in bold indicate signiﬁcant differences. Values in parentheses are in percentages. Normozoospermia (N ≥ 15 mln/mL of sperm cells); OS – oligozoospermia; Moderate OS (5 < N <15 mln/mL); Severe (1-5 mln/mL), very severe OS (N < 1 mln/mL); AS - azoospermia (lack of sperm cells in ejaculate); IVF, in vitro fertilization; P, probability; P_corr._, P x 4 – Bonferroni correction for multiple comparisons; OR, odds ratio; 95% CI, confidence interval from two-sided Fisher’s exact test; ns, not significant
