# Supplementary material for: KIR and HLA-C genes in male infertility
Source: J Assist Reprod Genet. 2020 May 20;37(8):2007–17. doi: 10.1007/s10815-020-01814-6 (PMC7467998; doi:10.1007/s10815-020-01814-6)
Supplement: Supplementary file 8 — (DOCX 21 kb) [file 10815_2020_1814_MOESM8_ESM.docx]

**Supplementary Table 7.** *KIR* gene frequencies in IVF men stratified according to sperm parameters

| **KIR** | **Normospermic men**  **N = 189** | **Men with abnormal sperm**  **N = 221** | **Asthenospermic men**  **N = 117** | **Teratospermic men**  **N = 28** | **Men with abnormal sperm vs.**  **Normospermic men** | | | **Asthenospermic men vs.**  **Normospermic men** | | | **Teratospermic men vs.**  **Normospermic men** | | |
| --- | --- | --- | --- | --- | --- | --- | --- | --- | --- | --- | --- | --- | --- |
|  |  |  |  |  | ***P*** | **OR** | **95%CI** | ***P*** | **OR** | **95%CI** | ***P*** | **OR** | **95%CI** |
| **2DL1** | 177 (93.65) | 211 (95.48) | 112 (95.73) | 27 (96.43) | 0.51 | 1.43 | 0.60-3.39 | 0.61 | 1.52 | 0.52-4.43 | 1.00 | 1.83 | 0.23-14.66 |
| **2DL2** | 110 (58.20) | 137 (61.99) | 77 (65.81) | 20 (71.43) | 0.48 | 1.17 | 0.79-1.74 | 0.23 | 1.38 | 0.86-2.23 | 0.22 | 1.80 | 0.75-4.28 |
| **2DL3** | 158 (83.60) | 195 (88.24) | 104 (88.89) | 27 (96.43) | 0.20 | 1.47 | 0.84-2.58 | 0.24 | 1.57 | 0.78-3.14 | 0.09 | 5.30 | 0.69-40.47 |
| **2DL4 norm** | 137 (72.49) | 154 (69.68) | 81 (69.23) | 19 (67.86) | 0.59 | 0.87 | 0.57-1.34 | 0.60 | 0.85 | 0.51-1.42 | 0.65 | 0.80 | 0.34-1.88 |
| **2DL4 del** | 141 (74.60) | 178 (80.54) | 95 (81.20) | 22 (78.57) | 0.15 | 1.41 | 0.88-2.25 | 0.21 | 1.47 | 0.83-2.59 | 0.82 | 1.25 | 0.48-3.26 |
| **2DL5 all** | 98 (51.85) | 113 (51.13) | 59 (50.43) | 15 (53.57) | 0.92 | 0.97 | 0.66-1.43 | 0.82 | 0.94 | 0.60-1.50 | 1.00 | 1.07 | 0.48-2.38 |
| **2DL5 gr.1** | 47 (24.87) | 62 (28.05) | 28 (23.93) | 8 (28.57) | 0.50 | 1.18 | 0.76-1.83 | 0.89 | 0.95 | 0.56-1.63 | 0.65 | 1.21 | 0.50-2.93 |
| **2DL5 gr.2** | 69 (36.51) | 78 (35.29) | 44 (37.61) | 11 (39.29) | 0.84 | 0.95 | 0.63-1.42 | 0.90 | 1.05 | 0.65-1.69 | 0.83 | 1.13 | 0.50-2.54 |
| **2DL5 exp** | 70 (37.04) | 82 (37.10) | 52 (44.44) | 8 (28.57) | 1.00 | 1.00 | 0.67-1.50 | 0.23 | 1.36 | 0.85-2.17 | 0.53 | 0.68 | 0.28-1.63 |
| **2DL5 null** | 76 (40.21) | 83 (37.56) | 48 (41.03) | 13 (46.43) | 0.61 | 0.89 | 0.60-1.33 | 0.91 | 1.03 | 0.65-1.65 | 0.54 | 1.29 | 0.58-2.86 |
| **2DS1** | 70 (37.04) | 87 (39.37) | 43 (36.75) | 12 (42.86) | 0.68 | 1.10 | 0.74-1.65 | 1.00 | 0.99 | 0.61-1.59 | 0.68 | 1.28 | 0.57-2.85 |
| **2DS2** | 109 (57.67) | 138 (62.44) | 77 (65.81) | 20 (71.43) | 0.36 | 1.22 | 0.82-1.82 | 0.19 | 1.41 | 0.88-2.28 | 0.22 | 1.84 | 0.77-4.38 |
| **2DS3** | 68 (35.98) | 77 (34.84) | 44 (37.61) | 11 (39.29) | 0.84 | 0.95 | 0.63-1.43 | 0.81 | 1.07 | 0.67-1.73 | 0.83 | 1.15 | 0.51-2.60 |
| **2DS4 norm** | 74 (39.15) | 76 (34.39) | 44 (37.61) | 8 (28.57) | 0.35 | 0.81 | 0.54-1.22 | 0.81 | 0.94 | 0.58-1.51 | 0.31 | 0.62 | 0.26-1.49 |
| **2DS4 del** | 151 (79.89) | 184 (83.26) | 98 (83.76) | 22 (78.57) | 0.44 | 1.25 | 0.76-2.07 | 0.45 | 1.30 | 0.71-2.38 | 0.81 | 0.92 | 0.35-2.44 |
| **2DS5** | 47 (24.87) | 61 (27.60) | 28 (23.93) | 8 (28.57) | 0.57 | 1.15 | 0.74-1.79 | 0.89 | 0.95 | 0.56-1.63 | 0.65 | 1.21 | 0.50-2.93 |
| **3DL1** | 177 (93.65) | 207 (93.67) | 112 (95.73) | 24 (85.71) | 1.00 | 1.00 | 0.45-2.22 | 0.61 | 1.52 | 0.52-4.43 | 0.13 | 0.41 | 0.12-1.36 |
| **3DL2** | 189 (100.0) | 220 (99.55) | 117 (100.0) | 27 (96.43) | 1.00 | 0.39 | 0.02-9.59 | - | - | - | 0.13 | 0.05 | 0.00-1.22 |
| **3DL3** | 189 (100.0) | 221 (100.0) | 117 (100.0) | 28 (100.0) | - | - | - | - | - | - | - | - | - |
| **3DS1** | 70 (37.04) | 82 (37.10) | 43 (36.75) | 9 (32.14) | 1.00 | 1.00 | 0.67-1.50 | 1.00 | 0.99 | 0.62-1.59 | 0.68 | 0.81 | 0.35-1.88 |
| **2DP1** | 178 (94.18) | 212 (95.93) | 113 (96.58) | 28 (100.0) | 0.49 | 1.46 | 0.59-3.59 | 0.42 | 1.75 | 0.54-5.62 | 0.37 | 3.67 | 0.21-64.10 |
| **3DP1** | 177 (93.65) | 211 (99.53) | 112 (95.73) | 27 (96.43) | 0.51 | 1.43 | 0.60-3.39 | 0.61 | 1.52 | 0.53-4.43 | 1.00 | 1.83 | 0.23-14.66 |
| **3DP1 var** | 62 (32.80) | 86 (38.91) | 48 (41.03) | 10 (35.71) | 0.22 | 1.31 | 0.87-1.96 | 0.18 | 1.43 | 0.88-2.30 | 0.83 | 1.14 | 0.50-2.61 |

Normospermia – total number of sperm cells, their concentration, progressive motility and morphology above or equal reference values; Men with abnormal sperm – men with at least one parameter of semen below reference value; Asthenospermia – number of sperm cells with progressive motility below reference values; Teratospermia – number of morphologically normal sperm cells below reference values; IVF, *in vitro* fertilization; *P*, probability; OR, odds ratio; 95% CI, confidence interval from two-sided Fisher’s exact test; Values in parentheses are in percentages.
