# Supplementary material for: KIR and HLA-C genes in male infertility
Source: J Assist Reprod Genet. 2020 May 20;37(8):2007–17. doi: 10.1007/s10815-020-01814-6 (PMC7467998; doi:10.1007/s10815-020-01814-6)
Supplement: Supplementary file 9 — (DOCX 18 kb) [file 10815_2020_1814_MOESM9_ESM.docx]

**Supplementary Table 8**. Centromeric and telomeric *KIR* genotypes in IVF men stratified according to sperm parameters

| **KIR genotype** | **Normospermic men**  **N = 189** | **Men with abnormal sperm**  **N = 221** | **Asthenospermic men**  **N = 117** | **Teratospermic men**  **N = 28** | **Men with abnormal sperm vs.**  **Normospermic men** | | | **Asthenospermic men**  **vs.**  **Normospermic men** | | | **Teratospermic men**  **vs.**  **Normospermic men** | | |
| --- | --- | --- | --- | --- | --- | --- | --- | --- | --- | --- | --- | --- | --- |
|  |  |  |  |  | ***P*/*P*_corr._** | **OR** | **95%CI** | ***P*/*P*_corr._** | **OR** | **95%CI** | ***P*/*P*_corr._** | **OR** | **95%CI** |
| **AA** | 52 (27.51) | 52 (23.53) | 30 (25.64) | 5 (17.86) | 0.36 | 0.81 | 0.52-1.27 | 0.79 | 0.91 | 0.54-1.53 | 0.36 | 0.57 | 0.21-1.59 |
| **Bx** | 137 (72.49) | 169 (76.47) | 87 (74.36) | 23 (82.14) | 0.36 | 1.23 | 0.79-1.93 | 0.79 | 1.10 | 0.65-1.86 | 0.36 | 1.75 | 0.63-4.84 |
|  |  |  |  |  |  |  |  |  |  |  |  |  |  |
| **Cen AA** | 77 (40.74) | 83 (37.56) | 40 (34.19) | 8 (28.57) | 0.54 | 0.87 | 0.59-1.30 | 0.28 | 0.76 | 0.47-1.22 | 0.3 | 0.58 | 0.24-1.39 |
| **Cen AB** | 81 (42.86) | 112 (50.68) | 64 (54.70) | 19 (67.86) | 0.14 | 1.37 | 0.93-2.03 | **0.046**/ns | **1.61** | **1.01-2.56** | **0.015**/ns | **2.82** | **1.21-6.55** |
| **Cen BB** | 31 (16.40) | 26 (11.76) | 13 (11.11) | 1 (3.57) | 0.20 | 0.68 | 0.39-1.19 | 0.24 | 0.64 | 0.32-1.28 | 0.09 | 0.19 | 0.02-1.44 |
| **Cen AB+Cen BB** | 112 (59.26) | 138 (62.44) | 77 (65.81) | 20 (71.43) | 0.54 | 1.14 | 0.77-1.70 | 0.28 | 1.32 | 0.82-2.14 | 0.30 | 1.72 | 0.72-4.10 |
|  |  |  |  |  |  |  |  |  |  |  |  |  |  |
| **Tel AA** | 112 (59.26) | 130 (58.82) | 71 (60.68) | 16 (57.14) | 1.00 | 0.98 | 0.66-1.46 | 0.81 | 1.06 | 0.66-1.70 | 0.84 | 0.92 | 0.41-2.05 |
| **Tel AB** | 66 (34.92) | 79 (35.75) | 41 (35.05) | 9 (32.14) | 0.92 | 1.04 | 0.69-1.56 | 1.00 | 1.01 | 0.62-1.63 | 0.83 | 0.88 | 0.38-2.06 |
| **Tel BB** | 11 (5.82) | 12 (5.43) | 5 (4.27) | 3 (10.72) | 1.00 | 0.93 | 0.40-2.16 | 0.61 | 0.72 | 0.25-2.14 | 0.40 | 1.94 | 0.51-7.44 |
| **Tel AB + Tel BB** | 77 (40.74) | 91 (41.18) | 46 (39.32) | 12 (42.86) | 1.00 | 1.02 | 0.69-1.51 | 0.81 | 0.94 | 0.59-1.51 | 0.84 | 1.09 | 0.49-2.44 |
|  |  |  |  |  |  |  |  |  |  |  |  |  |  |
| **Cen AA/Tel AA** | 52 (27.51) | 52 (23.53) | 30 (25.64) | 5 (17.86) | 0.36 | 0.81 | 0.52-1.27 | 0.79 | 0.91 | 0.54-1.53 | 0.36 | 0.57 | 0.21-1.59 |
| **Cen AA/Tel AB** | 24 (12.70) | 29 (13.12) | 10 (8.55) | 2 (7.14) | 1.00 | 1.04 | 0.58-1.85 | 0.35 | 0.64 | 0.30-1.40 | 0.54 | 0.53 | 0.12-2.37 |
| **Cen AA/Tel BB** | 1 (0.53) | 2 (0.91) | 0 (0.00) | 1 (3.57) | 1.00 | 1.72 | 0.15-19.10 | 1.00 | 0.53 | 0.02-13.25 | 0.24 | 6.96 | 0.42-114.70 |
| **Cen AB/Tel AA** | 41 (21.69) | 64 (28.96) | 35 (29.91) | 11 (39.29) | 0.11 | 1.47 | 0.94-2.31 | 0.13 | 1.54 | 0.91-2.61 | 0.056 | 2.34 | 1.02-5.38 |
| **Cen AB/Tel AB** | 33 (17.46) | 38 (17.19) | 24 (20.52) | 6 (21.43) | 1.00 | 0.98 | 0.59-1.64 | 0.55 | 1.22 | 0.68-2.19 | 0.61 | 1.29 | 0.48-3.43 |
| **Cen AB/Tel BB** | 7 (3.70) | 10 (4.52) | 5 (4.27) | 2 (7.14) | 0.81 | 1.23 | 0.46-3.30 | 0.77 | 1.16 | 0.36-3.75 | 0.33 | 2.00 | 0.39-10.15 |
| **Cen BB/Tel AA** | 19 (10.05) | 14 (6.33) | 6 (5.13) | 0 (0.00) | 0.20 | 0.61 | 0.29-1.24 | 0.14 | 0.48 | 0.19-1.25 | 0.14 | 0.15 | 0.01-2.61 |
| **Cen BB/Tel AB** | 9 (4.76) | 12 (5.43) | 7 (5.98) | 1 (3.57) | 0.83 | 1.15 | 0.47-2.79 | 0.79 | 1.27 | 0.46-3.52 | 1.00 | 0.74 | 0.09-6.08 |
| **Cen BB/Tel BB** | 3 (1.59) | 0 (0.00) | 0 (0.00) | 0 (0.00) | 0.10 | 0.12 | 0.00-2.35 | 0.29 | 0.23 | 0.01-4.43 | 1.00 | 0.93 | 0.05-18.59 |

Normospermia – total number of sperm cells, their concentration, progressive motility and morphology above or equal reference values; Men with abnormal sperm – men with at least one parameter of semen below reference value; Asthenospermia – number of sperm cells with progressive motility below reference values; Teratospermia – number of morphologically normal sperm cells below reference values; IVF, in vitro fertilization; *P*, probability; *P*_corr_., *P* x 4 – Bonferroni correction for multiple comparisons; OR, odds ratio; 95% CI, confidence interval from two-sided Fisher’s exact test; Values in bold indicate signiﬁcant differences; Values in parentheses are in percentages.
